# Increased Toll‐like Receptor‐MyD88‐NFκB‐Proinflammatory neuroimmune signaling in the orbitofrontal cortex of humans with alcohol use disorder

**DOI:** 10.1111/acer.14669

**Published:** 2021-08-20

**Authors:** Ryan P. Vetreno, Liya Qin, Leon G. Coleman, Fulton T. Crews

**Affiliations:** ^1^ Bowles Center for Alcohol Studies School of Medicine University of North Carolina at Chapel Hill Chapel Hill North Carolina USA; ^2^ Department of Psychiatry School of Medicine University of North Carolina at Chapel Hill Chapel Hill North Carolina USA; ^3^ Department of Pharmacology School of Medicine University of North Carolina at Chapel Hill Chapel Hill North Carolina USA

**Keywords:** cerebral cortex, chemokine, HMGB1, human, neurodegeneration

## Abstract

**Background:**

Many brain disorders, including alcohol use disorder (AUD), are associated with induction of multiple proinflammatory genes. One aspect of proinflammatory signaling is progressive increases in expression across cells and induction of other innate immune genes. High‐mobility group box 1 (HMGB1) heteromers contribute to amplification by potentiating multiple proinflammatory responses, including Toll‐like receptors (TLRs). TLR signaling recruits coupling proteins linked to nuclear transcription factors that induce proinflammatory cytokines and chemokines and their respective receptors. We tested the hypothesis that AUD induction of TLR expression increases levels of proinflammatory genes and cellular signaling cascades in association with neurodegeneration in the orbitofrontal cortex (OFC).

**Methods:**

Postmortem human OFC tissue samples (*n* = 10) from males diagnosed with AUD were compared to age‐matched moderate drinking controls (CON). Neuroimmune signaling molecules were assessed using immunohistochemistry for protein and reverse transcription polymerase chain reaction for messenger RNA (mRNA).

**Results:**

In the AUD OFC, we report induction of the endogenous TLR agonist HMGB1 as well as all TLRs assessed (i.e., TLR2‐TLR9) except TLR1. This was accompanied by increased expression of the TLR adaptor protein myeloid differentiation primary response 88 (MyD88), activation of the proinflammatory nuclear transcription factor nuclear factor kappa‐light‐chain‐enhancer of activated B cells (NFκB), and downstream induction of proinflammatory cytokines, chemokines, and their corresponding receptors. Several of these proinflammatory signaling markers are expressed in glia and neurons. The induction of HMGB1‐TLR‐MyD88‐NFκB proinflammatory signaling pathways correlates with neurodegeneration (i.e., Fluoro‐Jade B), lifetime alcohol consumption, and age of drinking onset.

**Conclusion:**

These data implicate the induction of HMGB1‐TLR‐MyD88‐NFκB cascades through coordinated glial and neuronal signaling as contributors to the neurodegeneration seen in the postmortem human OFC of individuals with AUD.

## Graphical abstract

Many brain disorders, including alcohol use disorder (AUD), are associated with induction of multiple proinflammatory genes and neurodegeneration. We tested the hypothesis that AUD induction of Toll‐like receptor (TLR) expression induces proinflammatory signaling cascades in association with neurodegeneration in the post‐mortem human orbitofrontal cortex (OFC). We report that induction of HMGB1‐TLR‐MyD88‐NFκB cascades through coordinated glial and neuronal signaling contributes to neurodegeneration in the OFC of individuals with AUD.

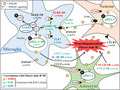

## INTRODUCTION

Neurodegeneration and neuroimmune signaling are associated with several disorders of the CNS, including Alzheimer's disease (AD) and related dementias, Parkinson's disease (PD), and alcohol use disorder (AUD) (Cheng et al., 2017; Crews et al., 2017; Van Hoesen et al., 2000; Hornberger et al., 2011; Kamal et al., 2020; Qin & Crews, 2012b). Neuroimmune signaling molecules of the innate immune system are expressed in the human brain, but their role in neurodegeneration is poorly understood. The orbitofrontal cortex (OFC), which is critically involved in regulating executive function (Schoenbaum et al., 2006), is vulnerable to damage in human AUD. Significant neuronal loss, volumetric reductions, and decreased connectivity are reported in the OFC of individuals with AUD and are accompanied by deficits in executive functioning (Crews & Boettiger, 2009; Miguel‐Hidalgo et al., 2006; Moorman, 2018; Qin & Crews, 2012b). Studies in alcohol‐treated rodents and postmortem humans diagnosed with AUD report decreased cortical neuron populations (Harper & Kril, 1989; Miguel‐Hidalgo et al., 2006) and increased markers of neuronal degeneration (Pascual et al., 2007; Qin & Crews, 2012b). Induction of proinflammatory neuroimmune signaling is thought to contribute to neurodegeneration (Crews & Vetreno, 2014; Glass et al., 2010; Kamal et al., 2020). Increased expression of neuroimmune signaling molecules is observed in the brains of alcohol‐treated rodents and postmortem humans with AUD (Crews et al., 2013; Erickson et al., 2019b; Guerri & Pascual, 2019; He & Crews, 2008), and *ex vivo* studies report that ethanol induces Toll‐like receptor (TLR) 7 signaling that can cause neuronal death (Coleman et al., 2017). However, the broader relationship between proinflammatory neuroimmune signals and neurodegeneration in the human AUD brain remains unclear.

Accumulating evidence implicates endogenous danger‐associated molecular patterns (DAMPs) and TLRs as factors contributing to neuroinflammation and neural degeneration across neurodegenerative disease states (Crews et al., 2017; Okun et al., 2009; Paudel et al., 2020). High‐mobility group box 1 (HMGB1), which is an endogenous cytokine‐like DAMP that signals across and regulates several TLRs, activates TLRs that signal through kinases to induce the proinflammatory nuclear transcription factor nuclear factor kappa‐light‐chain‐enhancer of activated B cells (NFκB). NFκB, in turn, induces transcription of proinflammatory cytokines, chemokines, and other genes associated with innate immune responses (Crews et al., 2011; Liu et al., 2017; Pahl, 1999). In rodent models, alcohol increases brain expression of HMGB1, TLRs, and proinflammatory genes through NFκB activation, which is accompanied by increased markers of neurodegeneration (Crews et al., 2006; McCarthy et al., 2018; Montesinos et al., 2016; Qin & Crews, 2012a; Vetreno & Crews, 2018; Vetreno et al., 2018). Further, treatment of mice with the inflammagen lipopolysaccharide persistently induces proinflammatory cytokines (e.g., tumor necrosis factor alpha [TNFα], interleukin‐1 beta [IL‐1β]) and chemokines (e.g., chemokine [C‐C motif] ligand 2 [CCL2]), which are accompanied by a delayed, progressive degeneration of substantia nigral dopaminergic neurons, similar to human PD (Qin et al., 2008; Qin et al., 2007). We find similarly increased expression of HMGB1, TLRs 2–4, and CCL2 as well as markers of neurodegeneration in the postmortem human AUD brain (Crews et al., 2013; He & Crews, 2008; Qin & Crews, 2012b). However, the association between neuroimmune signaling and neurodegeneration in human AUD is unknown. These findings led us to test the hypothesis that AUD‐associated induction of HMGB1 signaling through TLRs induces neuroimmune NFκB‐cytokine and chemokine signaling cascades that contribute to neurodegeneration in the human OFC.

## MATERIAL AND METHODS

### Human tissue

Postmortem human OFC paraffin‐embedded and frozen tissue samples from moderate drinking control (CON) and AUD subjects (*n* = 10/group) were obtained from the New South Wales Brain Tissue Resource Centre (NSW BTRC [Ethnics Committee Approval Number: X11‐0107]) at the University of Sydney (supported by the National Health and Medical Research Council of Australia‐Schizophrenia Research Institute and the National Institute of Alcohol Abuse and Alcoholism [NIH (NIAAA) R24AA012725]). Subject information was collected through personal interviews and next‐of‐kin interviews as well as medical records and is presented in Table 1. The NSW BTRC donor program uses a premortem consent program inviting members of the community to donate their postmortem brain tissue to neuroscience research, allowing collation and study of individuals with a wide variety of clinical histories of AUD and alcohol drinking. Since establishing an accurate alcohol drinking history and age of drinking onset is critical, trained clinical nurses and psychologists from the NSW BTRC performed extensive interviews with the human volunteers and their families. Alcohol drinking history and age of drinking onset information were derived from personal interviews with the volunteers as well as medical records and next‐of‐kin interviews. In cases where the age of drinking onset was unclear, an age of 25 was recorded (Sheedy et al., 2008). AUD subjects reported an average age of drinking onset of 16.6 (±0.5) years of age, which was compared to age‐matched CONs whose average age of drinking onset was 24.5 (±0.5). It is noteworthy that 100% of AUD subjects were able to recall their age of drinking onset, whereas only 10% of CONs were able to provide accurate information. Only individuals with AUD uncomplicated by liver cirrhosis and/or nutritional deficiencies were included in this study. All psychiatric and AUD diagnoses were confirmed using the Diagnostic Instrument for Brain Studies that complies with the Diagnostic and Statistical Manual of Mental Disorders (Dedova et al., 2009).

**TABLE 1:** Demographics of postmortem human moderate drinking control (CON) and alcohol use disorder (AUD) subjects

Classification	Age of Death	Brain Weight (g)	PMI (h)	Brain pH	RIN	Clinical Cause of Death	Age of Drinking Onset	Lifetime Alcohol Consumption (kg)	Years Drinking	BMI (kg/m^2^)
**CON**	**53**	**1590**	**16**	**6.8**	**7.9**	**Cardiac**	**25**	**102**	**28**	**26**
**CON**	**48**	**1330**	**24**	**6.7**	**6.9**	**Cardiac**	**25**	**17**	**23**	**24**
CON	44	1220	50	6.6	7.1	Cardiac	25	28	19	28
**CON**	**60**	**1420**	**28**	**6.8**	**8.0**	**Cardiac**	**25**	***unknown***	**35**	**29**
**CON**	**46**	**1320**	**29**	**6.1**	**4.4**	**Cardiac**	**25**	**115**	**21**	***unknown***
CON	24	1490	43	6.3	6.2	Cardiac	20	15	4	38
**CON**	**50**	**1426**	**30**	**6.4**	**7.5**	**Cardiac**	**25**	***unknown***	***unknown***	**28**
**CON**	**62**	**1430**	**46**	**7.0**	**8.8**	**Cardiac**	**25**	**5**	***7***	***33***
**CON**	**50**	**1596**	**40**	**6.9**	**8.6**	**Cardiac**	**25**	**18**	**25**	**29**
CON	40	1441	27	6.8	7.4	Cardiac	25	47	16	35
AUD	25	1400	43.5	6.7	6.9	Toxicity	16	552	9	19
**AUD**	**50**	**1520**	**17**	**6.3**	**7.0**	**Cardiac**	**18**	**2453**	**32**	**24**
AUD	44	1360	15	6.5	7.9	Cardiac	20	639	10	24
AUD	42	1400	41	6.5	8.0	Toxicity	18	1472	24	24
**AUD**	**45**	**1580**	**18.5**	**6.6**	**7.9**	**Respiratory**	**15**	**1799**	**29**	**29**
**AUD**	**61**	**1588**	**59**	**6.6**	**6.1**	**Cardiac**	**16**	**8052**	**43**	**25**
**AUD**	**49**	**1600**	**44**	**6.4**	**6.4**	**Cardiac**	**16**	**1012**	**33**	**26**
**AUD**	**49**	**1420**	**16**	**6.2**	**6.2**	**Cardiac**	**14**	**613**	**35**	**33**
**AUD**	**61**	**1340**	**23.5**	**6.9**	**8.3**	**Cardiac**	**17**	**5621**	**44**	**25**
AUD	50	1470	34.5	6.9	7.3	Respiratory	16	5212	34	28

Notes

Age of drinking onset was significantly different (*t*(18) = −10.7, *p* = 0.0001) between CON (24.5 ± 0.5) and AUD subjects (16.6 ±0.5). Lifetime alcohol consumption (CON: 39 kg ±14, AUD: 2742 kg ±829; *t*(9.0) = 3.3, *p* = 0.010, Welch's *t* test) and body mass index (BMI; CON: 30 kg/m^2^ ±1, AUD: 26 kg/m^2^ ±1; *t*(17) = −2.3, *p* = 0.035) were significantly different between groups. No differences were observed regarding age of death (*p* = 0.98), brain weight (*p* = 0.40), postmortem interval (PMI; *p* = 0.73), brain pH (*p* = 0.50), RNA integrity number (RIN; *p* = 0.87), or years drinking (*p* = 0.08). Bold denotes CON (*n* = 7) and AUD (*n* = 6) individuals from a prior study (Qin & Crews, 2012b) that overlapped with subjects in the current study used for Fluoro‐Jade B correlation with neuroimmune markers. Within this cohort, age of drinking onset (*t*(5.0) = −15.6, *p* = 0.00002, Welch's *t* test) differed significantly between CON and AUD subjects. Lifetime alcohol consumption (*t*(5.0) = 2.7, *p* = 0.044, Welch's *t* test) and total years drinking (*t*(10) = 2.8, *p* = 0.020) were also significantly different between groups. No differences were observed regarding age of death (*p* = 0.95), brain weight (*p* = 0.32), PMI (*p* = 0.92), brain pH (*p* = 0.29), RIN (*p* = 0.53), or BMI (*p* = 0.55).

### RNA extraction and reverse transcription PCR (RT‐PCR)

Total RNA was extracted from frozen human OFC tissue samples from CON and AUD subjects by homogenization in TRI reagent (Sigma‐Aldrich) following the single‐step method of RNA isolation (Chomczynski & Sacchi, 2006). RNA quality and concentration was determined using a NanoDrop 1000 (Thermo Fisher Scientific). Total mRNA was reverse transcribed as previously described (Vetreno et al., 2018). RT‐PCRs were run on a Bio‐Rad CFX system (Bio‐Rad). SYBER Green PCR Master Mix (Life Technologies) was used for RT‐PCR. RT‐PCR was run with an initial activation for 10 min at 95°C, followed by 40 cycles of denaturation (95°C, 15 s), annealing/extension (57–58°C, 1 min), and melt curve. The primer sequences are presented in Table S1. Differences in primer expression between groups are expressed as cycle time (Ct) values normalized with β‐actin, and relative differences between groups calculated and expressed as the percent difference relative to CONs.

### Immunohistochemistry

Paraffin‐embedded postmortem human OFC sections were deparaffinized, washed in PBS, and antigen retrieval performed by incubation in Citra solution (BioGenex) for 1 h at 70°C. Following incubation in blocking solution (MP Biomedicals), slides were incubated in a primary antibody solution for 24 h at 4°C. Primary antibodies, dilutions, and validation information are included in Table S2. Slides were incubated for 1 h in biotinylated secondary antibody (1:200; Vector Laboratories) and then for 1 h in avidin–biotin complex solution (Vector Laboratories). The chromogen nickel‐enhanced diaminobenzidine (Sigma‐Aldrich) was used to visualize immunoreactivity. Slides were dehydrated and cover slipped. Negative control for nonspecific binding was conducted employing the above‐mentioned procedures with omission of the antibody.

### Microscopic quantification

BioQuant Nova Advanced Image Analysis software (R&M Biometric) was used for image capture and quantification of immunohistochemistry. Representative images were captured using an Olympus BX50 microscope and Sony DXC‐390 video camera linked to a computer. For each measure, the microscope, camera, and software were background corrected and normalized to preset light levels to ensure fidelity of data acquisition. A modified unbiased stereological quantification method was used to quantify TLR9‐, MyD88‐, IKKβ‐, cleaved IL‐1β‐, CCL2‐, and CXCL8‐immunoreactive (+IR) cells in the postmortem human OFC. We previously reported that comparison of traditional unbiased stereological methodology with our modified unbiased stereological approach yielded nearly identical values relative to control subjects (Crews et al., 2004). The outlined regions of interest were determined and data expressed as cells/mm^2^.

### Fluoro‐Jade B immunofluorescent staining

Fluoro‐Jade B immunofluorescent data were used from a previously published study (Qin & Crews, 2012b). Briefly, paraffin‐embedded human OFC sections were deparaffinized, washed in PBS, and immunostained with mouse anti‐NeuN (1:100; Millipore). Immunolabeling was visualized using Alexa Fluor 555 dye. Sections were washed in PBS and water, then transferred to a solution of 0.06% potassium permanganate for 10 min. Sections were then rinsed in distilled water for 2 min and placed in a 0.0004% Fluoro‐Jade B solution made by adding 4.0 ml of a 0.01% stock solution of Fluoro‐Jade B (Millipore) to 96 ml of 0.1% acetic acid. After 20 min in the Fluoro‐Jade B solution, stained slides were thoroughly washed in distilled water, dehydrated, and cover slipped (Qin & Crews, 2012b). Fluorescent intensity of Fluoro‐Jade B was quantified using BioQuant Nova Advanced Image Analysis software.

### Fluorescent immunohistochemistry and microscopy

Paraffin‐embedded human OFC sections were deparaffinized, washed in PBS, and antigen retrieval performed by incubation in Citra solution (BioGenex) for 1 h at 70°C. Following incubation in blocking solution (MP Biomedicals), slides were incubated for 24 h at 4°C in a primary antibody solution consisting of blocking solution or Dako antibody diluent (Dako, Denmark) in combination with either rabbit anti‐TLR9 (1:70), rabbit anti‐pNF‐κB p65 (phospho S536) (1:150), mouse anti‐MCP‐1 (1:100), or mouse anti‐IL‐8 (1:300) combined with an antibody against neurons (chicken anti‐NeuN; 1:100; Novus; Cat. #NBP2‐10491), astrocytes (mouse anti‐GFAP; 1:50; Abcam; Cat. #ab4648 or rabbit anti‐GFAP; 1:250; Dako, Denmark; Cat. #Z0334), or microglia (mouse anti‐Iba‐1; 1:250; Wako, Osaka, Japan; Cat. #019‐19741). Slides were washed in PBS and incubated for 1 h at room temperature with the appropriate secondary antibodies (1:1000). Immunoblotting was visualized using Alexa Fluor 488 or 594 dye. Secondary‐only negative controls were performed without primary antibody incubation. Slides were coverslipped using Prolong Gold Anti‐fade mounting media (Life Technologies). Immunofluorescent images were obtained using a Nikon DS‐Ri2 scope (Nikon Inc.) and colocalization quantified using NIS Elements AR46 (Nikon Inc.).

### Statistical analysis

Statistical analysis was performed using SPSS. Sample size determinations were based on previously published studies (Crews et al., 2013; Liu et al., 2020). Two‐tailed Student's *t* tests were used to assess human demographics, RT‐PCR, and immunohistochemistry data unless otherwise reported. Levene's Test for Equality of Variances was performed for each analysis. When reported in the Results, Welch's *t* tests were used to assess data with unequal variances. Two‐tailed Pearson's *r* was used for all correlative analyses. Since performing multiple comparisons can increase the incidence of Type I Errors, the Benjamini‐Hochberg procedure (B‐H Critical) for controlling false positives was calculated (Thissen et al., 2002) for neuroimmune correlations with Fluoro‐Jade B immunohistochemistry. An individual Pearson's *r* correlation was considered statistically significant if the *p* value was less than the B‐H Critical value (false discovery rate threshold = 0.1). All values are reported as mean ± SEM, and significance was defined as *p* ≤ 0.05.

## RESULTS

### Induction of TLRs and HMGB1 signaling genes in the postmortem OFC of individuals with AUD

**FIGURE 1:**
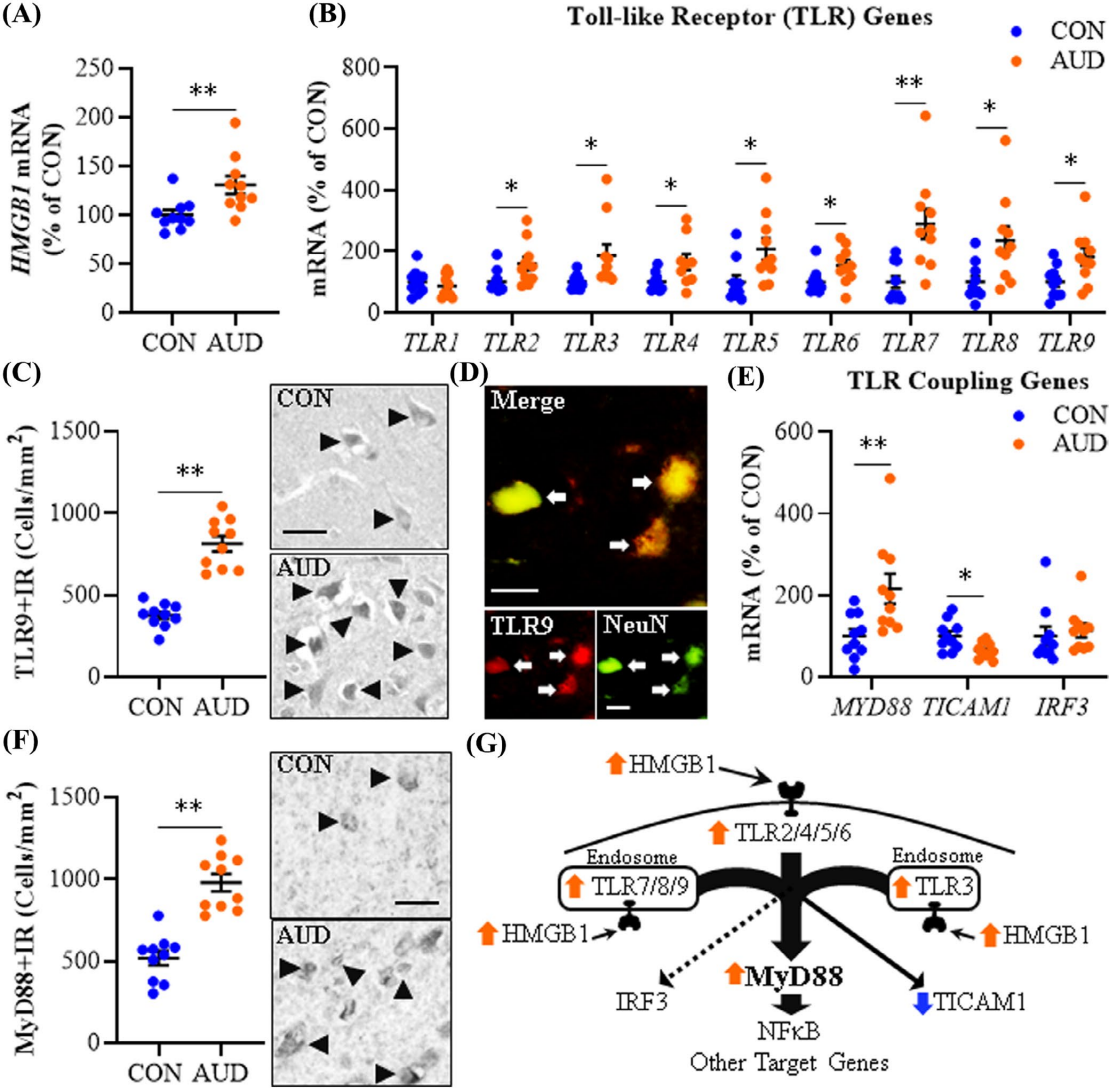
Expression of Toll‐like receptors (TLRs) and high‐mobility group box 1 (HMGB1) in age‐matched moderate drinking control (CON) and alcohol use disorder (AUD) postmortem human orbitofrontal cortex (OFC). (A) Reverse transcription PCR (RT‐PCR) analysis revealed AUD increased *HMGB1* expression approximately 1.4‐fold relative to CONs. (B) RT‐PCR analysis revealed AUD increased expression of *TLR2* (1.6‐fold), *TLR3* (1.9‐fold), *TLR4* (1.5‐fold), *TLR5* (2.1‐fold), *TLR6* (1.5‐fold), *TLR7* (2.9‐fold), *TLR8* (2.4‐fold), and *TLR9* (1.8‐fold) relative to CONs. (C) Modified unbiased stereological assessment revealed a 2.2‐fold increase of TLR9+IR in the AUD OFC relative to CONs. Representative photomicrographs of TLR9+IR in the OFC of a CON and AUD sample. Black arrowheads indicate TLR9+IR cells. Scale bar = 30 μm. (D) Immunofluorescent co‐labeling revealed a high degree of TLR9 (red) colocalization with the neuronal marker NeuN (green) in the postmortem human OFC. White arrows indicate TLR9+IR cells that colocalize with NeuN (top; yellow). Scale bar = 20 μm. (E) RT‐PCR analysis revealed AUD increased expression of *MYD88* (2.2‐fold) and decreased expression of *TICAM1* (TRIF; 35% ±6%) relative to CONs. (F) Modified unbiased stereological assessment revealed an approximate 2‐fold increase of MyD88+IR in the AUD OFC relative to CONs. Representative photomicrographs of MyD88+IR in the OFC of a CON and AUD sample. Black arrowheads indicate TLR9+IR cells. Scale bar = 30 μm. (G) Simplified schematic depicting HMGB1 signaling through TLRs and TLR coupling adaptors to NF‐κB and other target genes. RT‐PCR analyses were run in triplicate. Data are presented as mean ±SEM. * *p* ≤ 0.05, ** *p* < 0.01

Toll‐like receptors, originally characterized as receptors for exogenous pathogens, have emerged as neuroimmune signaling receptors in the sterile brain that respond to endogenous agonists, thereby inducing self‐expression and amplifying signals across cells through autocrine and paracrine mechanisms. In previous studies, we reported significantly increased protein levels of TLR2, TLR3, TLR4, and TLR7 as well as the endogenous TLR agonist HMGB1 in the AUD brain (Coleman et al., 2017; Crews et al., 2013; Qin et al., 2021). In the present investigation, we assessed induction of the universal TLR agonist HMGB1 and its association with levels of TLRs 1–9 in the postmortem human OFC of individuals diagnosed with AUD (*n* = 10) relative to age‐matched CONs (*n* = 10). Individuals with AUD averaged lifetime alcohol consumption levels of 2663 kg of alcohol, whereas CONs averaged 39 kg of lifetime alcohol consumption (Table 1). Postmortem brain weights did not differ significantly between CON (1426 g ±37) and AUD (1468 g ±31) individuals. Expression of *HMGB1* mRNA increased 1.4‐fold (*t*(18) = 2.9, *p* = 0.009) in the AUD OFC relative to CONs (Figure 1A). Determination of TLR gene expression revealed an AUD‐induced increase of *TLR2* (1.6‐fold; *t*(18) = 2.4, *p* = 0.029), *TLR3* (1.9‐fold; *t*(9.8) = 2.4, *p* = 0.038, Welch's *t* test), *TLR4* (1.5‐fold; *t*(16) = 2.3, *p* = 0.035), *TLR5* (2.1‐fold; *t*(18) = 2.6, *p* = 0.018), *TLR6* (1.5‐fold; *t*(18) = 2.4, *p* = 0.027), *TLR7* (2.9‐fold; *t*(18) = 3.6, *p* = 0.002), *TLR8* (2.4‐fold; *t*(18) = 2.7, *p* = 0.013), and *TLR9* (1.8‐fold; *t*(18) = 2.5, *p* = 0.023) relative to CONs (Figure 1B). *TLR1* was unchanged between groups. *HMGB1* expression across subjects significantly correlated with *TLR2* and TLRs *4*–*9*, but not *TLR3* (Table S3). Further, expression of each *TLR* positively correlated with expression of all other TLRs, except *TLR3* and *TLR4*. For example, *TLR9* expression was positively correlated with *HMGB1*, *TLR2*, and *TLR*s *5*–*8*, but not *TLR3* or *TLR4*, consistent with HMGB1‐TLR activation inducing expression of additional TLRs. TLR9 is a nucleic acid‐sensing TLR activated by HMGB1‐CpG oligonucleotide complexes known to be particularly responsive to unmethylated CpG sequences common in viruses (Martinez‐Campos et al., 2017). Immunohistochemical determination of TLR9 cellular protein in the OFC revealed an AUD‐induced approximate 2.2‐fold increase of TLR9+IR cells (*t*(13.1) = 8.3, *p* = 0.000001, Welch's *t* test) relative to CONs (Figure 1C). Colocalization of TLR9+IR cells with Iba‐1 for microglia, GFAP for astrocytes (Figure S1A,B), and NeuN for neurons (Figure 1D) revealed co‐expression across cell types. Together, these data reveal that AUD increases expression of HMGB1 and most TLR subtypes in the postmortem human OFC with significant associations of HMGB1 levels with expression of several TLRs.

Agonist‐activated TLRs couple to the intracellular signaling molecules myeloid differentiation primary response 88 (MyD88) and TIR‐domain‐containing adapter‐inducing interferon‐β (TRIF [*TICAM1*]) as well as the viral‐mediated TLR adaptor interferon regulatory factor 3 (IRF3). We report here that AUD increased *MYD88* mRNA expression approximately 2.2‐fold (*t*(18) = 2.9, *p* = 0.010), but decreased *TICAM1* by 35% (±6%; *t*(18) = −2.8, *p* = 0.013) in the OFC relative to CONs (Figure 1E). *IRF3*, a downstream transcription factor for TLR3, was unchanged between groups. Immunohistological determination of MyD88 cellular protein revealed an AUD‐induced approximate 2‐fold increase of MyD88+IR cells (*t*(18) = 6.7, *p* = 0.000003) relative to CONs (Figure 1F). MyD88 is a universal TLR adaptor protein, except at TLR3, and increased expression of MyD88 suggests increased TLR‐MyD88 signaling in AUD. *MYD88* expression across subjects significantly correlated with *HMGB1*, *TLR2*, and *TLR*s *5*–*9*, but not *TLR3* or *TLR4* (Table S3). For example, *TLR9* showed a strong positive correlation with *MYD88*. In contrast, *TICAM1* negatively correlated with *HMGB1*, *TLR2*, *TLR5*, and *TLR6*. These findings suggest that AUD increases HMGB1‐TLR signaling linked to induction of MyD88 and reduced TICAM1 in the human OFC (Figure 1G).

### Increased expression of NFκB family and signaling genes in the postmortem OFC of individuals with AUD

**FIGURE 2:**
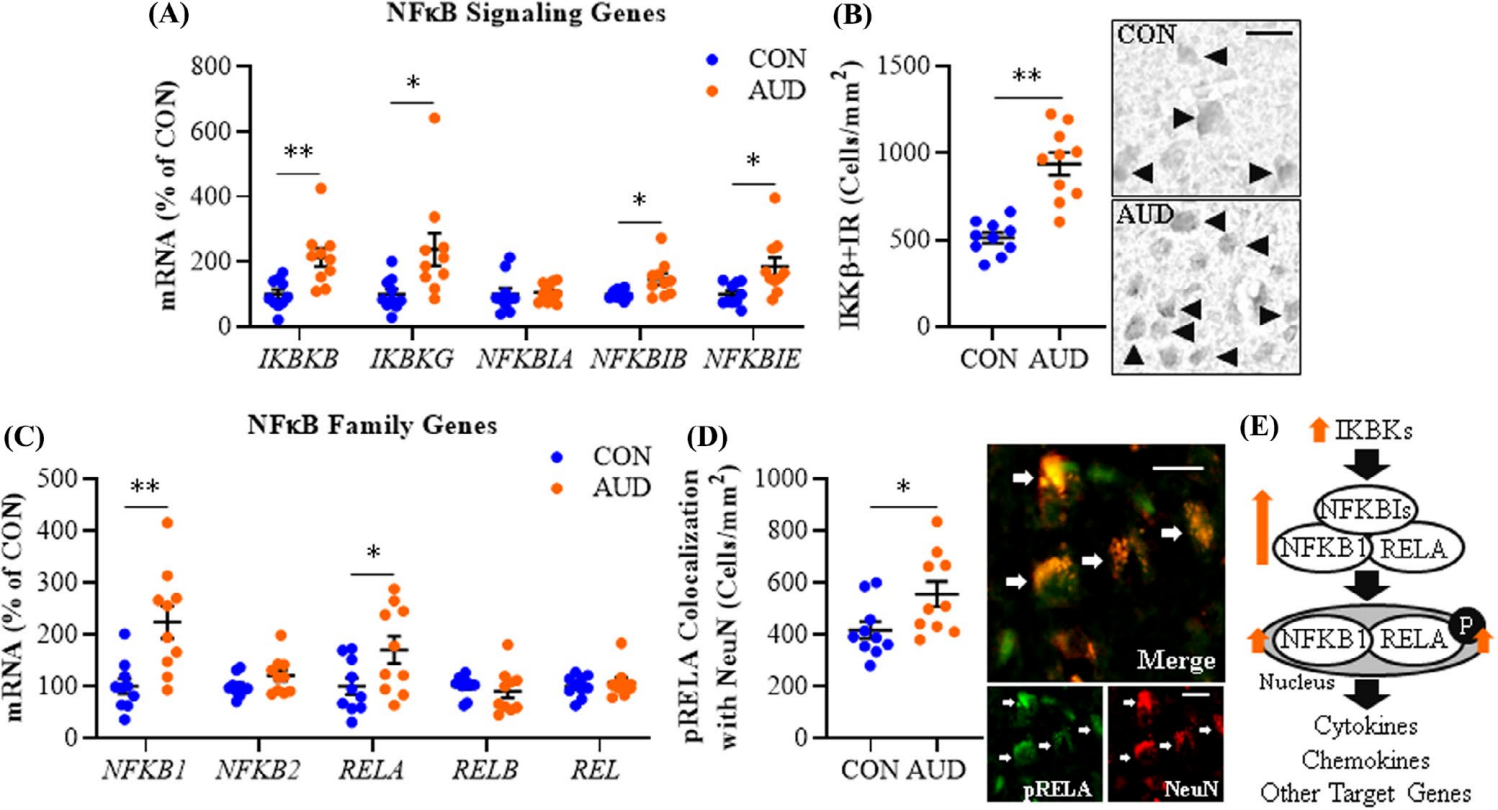
Expression of NFκB signaling molecules in age‐matched moderate drinking control (CON) and alcohol use disorder (AUD) postmortem human orbitofrontal cortex (OFC). (A) Reverse transcription PCR (RT‐PCR) analysis revealed AUD increased expression of *IKBKB* (IKKβ; 2.1‐fold), *IKBKG* (2.4‐fold), *NFKBIB* (1.5‐fold), and *NFKBIE* (1.9‐fold) relative to CONs. (B) Modified unbiased stereological assessment revealed an approximate 1.8‐fold increase of IKKβ+IR in the AUD OFC relative to CONs. Representative photomicrographs of IKKβ+IR in the OFC of a CON and AUD sample. Black arrowheads indicate IKKβ+IR cells. Scale bar = 30 μm. (C) RT‐PCR analysis revealed AUD increased expression of *NFKB1* (2.2‐fold) and *RELA* (1.7‐fold) relative to CONs. (D) Immunofluorescent co‐labeling revealed a high degree of phosphorylated (activated) RELA (pRELA; green) colocalization with the neuronal marker NeuN (red) in the postmortem human OFC. White arrows indicate pRELA+IR cells that colocalize with NeuN (top; yellow). Scale bar = 20 μm. (E) Simplified schematic depicting IκΒ kinase complex phosphorylating and degrading IκΒs (inhibitor of nuclear factor kappa B) allowing nuclear translocation and activation of the NFκB complex. RT‐PCR analyses were run in triplicate. Data are presented as mean ± SEM. * *p* ≤ 0.05, **p < 0.01

Preclinical studies find alcohol induces and releases HMGB1 that activates TLRs and other neuroimmune receptors, increasing transcription of NFκB proinflammatory genes, including TLRs, within and across cells (Coleman & Crews, 2018; Crews et al., 2017). We assessed expression of genes within the NFκB transcription factor family, including transcriptional activators (i.e., IκΒ kinase [IKK] complex [*IKBK*s]) and regulatory inhibitors (i.e., IκΒs [*NFKBI*s]). We report an approximate 2.1‐fold increase of *IKBKB* (IKKβ; *t*(18) = 3.5, *p* = 0.002) and 2.4‐fold increase of *IKBKG* (*t*(18) = 2.6, *p* = 0.018) that was accompanied by a 1.5‐fold increase of *NFKBIB* (*t*(10.2) = 2.6, *p* = 0.025, Welch's *t* test) and a 1.9‐fold increase of *NFKBIE* (*t*(18) = 2.8, *p* = 0.012) in AUD relative to CONs (Figure 2A). *NFKBIA* was unchanged between groups. NFκB activation induces expression of IKKs as well as IκΒs, consistent with increases in regulatory repressor NFκB signaling genes being induced by HMGB1‐TLR‐MyD88 signaling. Immunohistological assessment of IKKβ cellular protein revealed an AUD‐induced approximate 1.8‐fold increase of IKKβ+IR cells (*t*(12.6) = 5.9, *p* = 0.00006, Welch's *t* test) relative to CONs (Figure 2B). NFκB transcriptional activation involves the canonical NFκB family members *NFKB1* and *RELA* that form a heterodimer that activates proinflammatory gene transcription. We further found AUD induced an approximate 2.2‐fold increase of *NFKB1* (*t*(12.8) = 3.6, *p* = 0.003, Welch's *t* test) and a 1.7‐fold increase of *RELA* (*t*(14.8) = 2.3, *p* = 0.039; Welch's *t* test) relative to CONs (Figure 2C). *NFKB2*, *RELB*, and *REL* were unchanged between groups. Increases of *NFKB1* and *RELA* are consistent with activation of NFκB transcription, and we previously reported increased activated pRELA+IR in the postmortem human AUD OFC relative to CONs (Qin et al., 2021). We extend those findings with colocalization of pRELA+IR with Iba‐1+IR microglia, GFAP+IR astrocytes (Figure S1C,D), and NeuN+IR neurons (Figure 2D), consistent with NFκB activation across cell types within the AUD OFC. Expression of *HMGB1*, most *TLR*s, and *MYD88* correlated with all induced NFκB genes with the surprising exception of *TLR3* and *TLR4* (Table S4). For example, *TLR9* showed a robust positive correlation with both *HMGB1* and *MYD88* as well as all induced NFκB signaling genes. These findings are consistent with AUD increasing HMGB1‐TLR‐MyD88 expression that is coordinated with induction of NFκB subunit expression and activation of the proinflammatory nuclear transcription factor NFκB across glia and neurons (Figure 2E).

### Increased expression of cytokine signaling genes in the postmortem OFC of individuals with AUD

**FIGURE 3:**
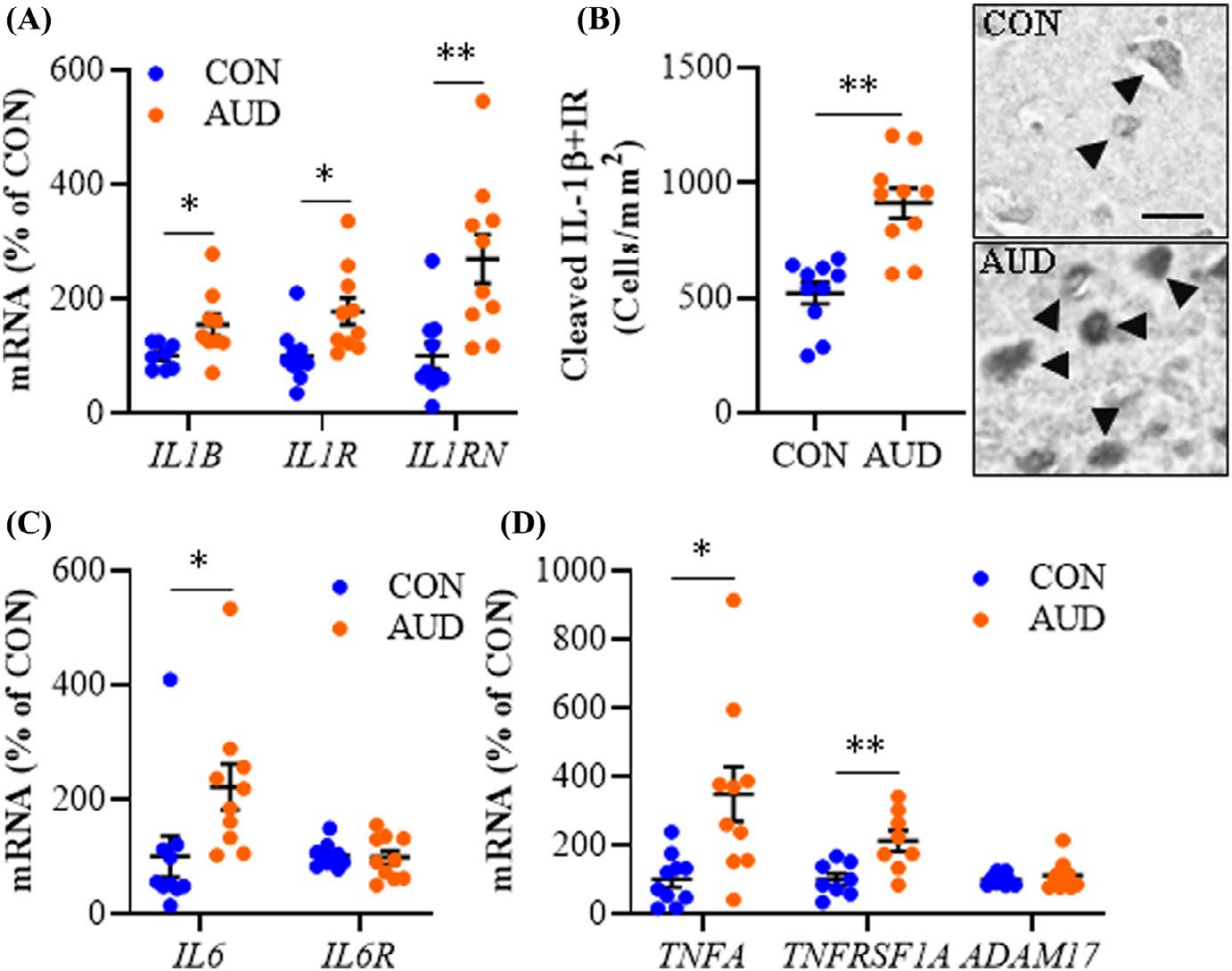
Expression of proinflammatory cytokines in age‐matched moderate drinking control (CON) and alcohol use disorder (AUD) postmortem human orbitofrontal cortex (OFC). (A) Reverse transcription PCR (RT‐PCR) analysis revealed AUD increased expression of *IL1B* (1.6‐fold), *IL1R* (1.8‐fold), and *IL1RN* (2.7‐fold) relative to CONs. (B) Modified unbiased stereological assessment revealed an approximate 1.8‐fold increase of cleaved IL‐1β+IR in the AUD OFC relative to CONs. Representative photomicrographs of IL‐1β+IR in the OFC of a CON and AUD sample. Black arrowheads indicate IL‐1β+IR cells. Scale bar = 30 μm. (C) RT‐PCR analysis revealed AUD increased expression of *IL6* (2.2‐fold) relative to CONs. (D) RT‐PCR analysis revealed AUD increased expression of *TNFA* (3.5‐fold) and *TNFRSF1A* (2.1‐fold) relative to CONs. RT‐PCR analyses were run in triplicate. Data are presented as mean ±SEM. * *p *≤ 0.05, **p < 0.01

Proinflammatory cytokines and their accompanying receptors are induced by and induce HMGB1‐TLR‐MyD88‐NFκB signaling cascades. We report AUD induced an approximate 1.6‐fold increase of *IL1B* (*t*(14) = 2.5, *p* = 0.028) and a 1.8‐fold increase of the IL1β receptor *IL1R* (*t*(18) = 2.8, *p* = 0.012) relative to CONs. This was accompanied by an approximate 2.7‐fold increase in expression of the IL1 receptor antagonist *IL1RN* (*t*(18) = 3.5, *p* = 0.003) in the AUD OFC (Figure 3A). Immunohistochemical assessment of cleaved IL‐1β cellular protein revealed an approximate 1.8‐fold increase in the AUD OFC (*t*(18) = 4.9, *p* = 0.0001) relative to CONs (Figure 3B). *IL6* was also increased by approximately 2.2‐fold (*t*(18) = 2.3, *p* = 0.036) in the AUD OFC relative to moderate drinking CONs, whereas expression of *IL6R* was unchanged (Figure 3C). Expression of *TNFA* was increased approximately 3.5‐fold (*t*(10.5) = 3.0, *p* = 0.013, Welch's *t* test), which was accompanied by a 2.1‐fold increase in expression of the TNFα receptor *TNFRSF1A* (*t*(14) = 3.2, *p* = 0.007) in the AUD OFC relative to CONs (Figure 3D). *ADAM17* was unchanged in the AUD OFC. All TLRs and *MYD88*, except *TLR3* and *TLR4*, showed robust positive correlations with *IL1R*, *IL1RN*, *IL6*, and *TNFRSF1A*, but not *IL1B* or *TNFA*, suggesting independent mechanisms of AUD gene induction (Table S5). For example, *TLR9*, which robustly correlated with *HMGB1*, *MYD88*, and induced NFκB signaling genes, also strongly correlated with *IL1R*, *IL1RN*, *IL6*, and *TNFRSF1A*, but not *IL1B* or *TNFA*. The significant and strong correlations of HMGB1‐TLR‐MyD88‐NFκB signaling cascades with *IL6*, *IL1R*, *IL1RN*, and *TNFRSF1A* are consistent with a coordinated TLR‐induced neuroimmune response in the OFC.

### Increased expression of chemokine and chemokine receptor genes in the postmortem OFC of individuals with AUD

**FIGURE 4:**
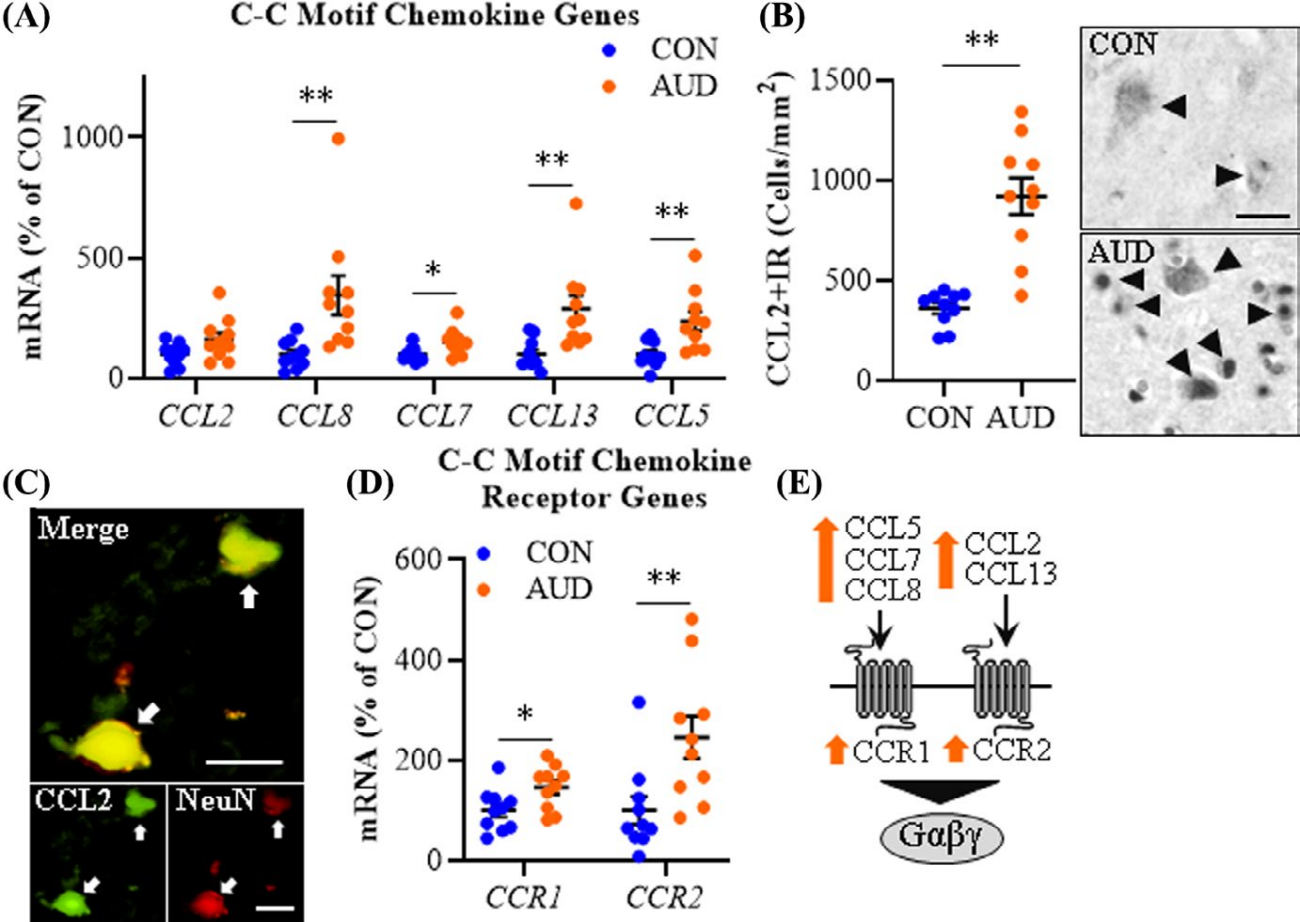
Expression of C‐C motif chemokine and receptors in age‐matched moderate drinking control (CON) and alcohol use disorder (AUD) postmortem human orbitofrontal cortex (OFC). (A) Reverse transcription PCR (RT‐PCR) analysis revealed AUD increased expression of *CCL8* (3.5‐fold), *CCL7* (1.5‐fold), *CCL13* (2.9‐fold), and *CCL5* (2.4‐fold) relative to CONs. (B) Modified unbiased stereological assessment revealed an approximate 2.6‐fold increase of CCL2+IR in the AUD OFC relative to CONs. Representative photomicrographs of CCL2+IR in the OFC of a CON and AUD sample. Black arrowheads indicate CCL2+IR cells. Scale bar = 30 μm. (C) Immunofluorescent co‐labeling revealed a high degree of CCL2 (green) colocalization with the neuronal marker NeuN (red) in the postmortem human OFC. White arrows indicate CCL2+IR cells that colocalize with NeuN (top; yellow). Scale bar = 20 μm. (D) RT‐PCR analysis revealed AUD increased expression of *CCR1* (1.5‐fold) and *CCR2* (2.5‐fold) relative to CONs. (E) Simplified schematic depicting C‐C motif chemokines interacting with C‐C receptors. RT‐PCR analyses were run in triplicate. Data are presented as mean ±SEM. * *p* ≤ 0.05, **p < 0.01

We previously reported that AUD increased CCL2 protein expression in the postmortem human hippocampus, amygdala, VTA, and substantia nigra (He & Crews, 2008), and CXCL8 protein expression in the hippocampus (Liu et al., 2020). In the present study, we investigated expression of multiple chemokines, including the C‐C motif chemokine (β‐chemokine) and C‐X‐C motif chemokine (α‐chemokine) families, and their associated G‐protein‐coupled receptors in the postmortem human OFC. In AUD, we report an approximate 3.5‐fold increase of *CCL8* (*t*(18) = 3.0, *p* = 0.008), a 1.5‐fold increase of *CCL7* (*t*(18) = 2.7, *p* = 0.016), a 2.9‐fold increase of *CCL13* (*t*(18) = 3.2, *p* = 0.005), and a 2.4‐fold increase of *CCL5* (*t*(18) = 3.2, *p* = 0.005) in the AUD individuals relative to CONs (Figure 4A). Expression of *CCL2* approached significance (*t*(18) = 2.0, *p* = 0.059), whereas *CCL3*, *CCL4*, and *CCL19* expression was unchanged between groups. Immunohistological assessment of CCL2 cellular protein expression revealed an approximate 2.6‐fold increase in the AUD OFC (*t*(5.8) = 10.6, *p* = 0.0001, Welch's *t* test) relative to CONs (Figure 4B). Immunohistological assessment of CCL2 protein revealed colocalization with Iba‐1+IR microglia, GFAP+IR astrocytes (Figure S1E,F), and NeuN+IR neurons (Figure 4C). Further, expression of β‐chemokine receptors *CCR1* (*t*(18) = 2.4, *p* = 0.026) and *CCR2 (t*(18) = 2.9, *p* = 0.010) was increased approximately 1.5‐fold and 2.5‐fold, respectively, in the AUD OFC relative to CONs (Figure 4D), consistent with AUD induction of C‐C motif chemokines and their receptors (Figure 4E).

**FIGURE 5:**
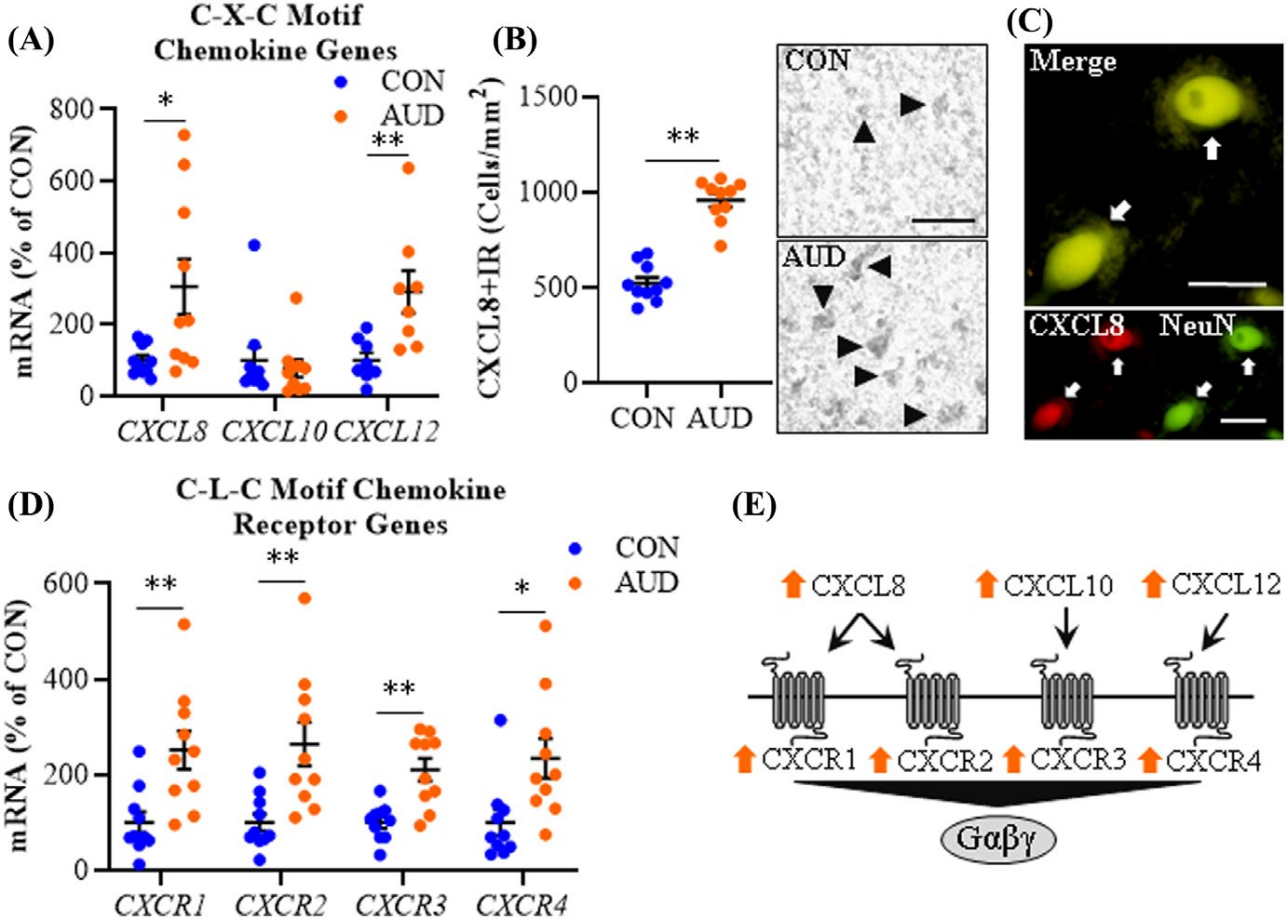
Expression of C‐X‐C motif chemokine and receptors in age‐matched moderate drinking control (CON) and alcohol use disorder (AUD) postmortem human orbitofrontal cortex (OFC). (A) Reverse transcription PCR (RT‐PCR) analysis revealed AUD increased expression of *CXCL8* (3‐fold) and *CXCL12* (2.9‐fold) relative to CONs. (B) Modified unbiased stereological assessment revealed an approximate 1.8‐fold increase of CXCL8+IR in the AUD OFC relative to CONs. Representative photomicrographs of CXCL8+IR in the OFC of a CON and AUD sample. Black arrowheads indicate CXCL8+IR cells. Scale bar = 50 μm. (C) Immunofluorescent co‐labeling revealed a high degree of CXCL8 (red) colocalization with the neuronal marker NeuN (green) in the postmortem human OFC. White arrows indicate CXCL8+IR cells that colocalize with NeuN (top; yellow). Scale bar = 20 μm. (D) RT‐PCR analysis revealed AUD increased expression of *CXCR1* (2.5‐fold), *CXCR2* (2.6‐fold), *CXCR3* (2.1‐fold), and *CXCR4* (2.4‐fold) relative to CONs. (E) Simplified schematic depicting C‐X‐C motif chemokines interacting with C‐X‐C motif chemokine receptors. RT‐PCR analyses were run in triplicate. Data are presented as mean ±SEM. * *p* ≤ 0.05, ** *p* < 0.01

Assessment of C‐X‐C motif chemokines revealed an AUD‐induced approximate 3‐fold increase of *CXCL8* (*t*(9.5) = 2.6, *p* = 0.026, Welch's *t* test) and a 2.9‐fold increase of *CXCL12* (*t*(14) = 3.1, *p* = 0.009) in the AUD OFC relative to CONs (Figure 5A). *CXCL10* was unchanged across groups. Immunohistological assessment of CXCL8 cellular protein revealed an approximate 1.8‐fold increase in the AUD OFC (*t*(18) = 9.4, *p* = 0.00000001) relative to CONs (Figure 5B). We observed colocalization of CXCL8 with Iba‐1+IR microglia, GFAP+IR astrocytes (Figure S1G,H), and NeuN+IR neurons (Figure 5C) in the OFC. In humans, CXCL8 has 2 receptors, *CXCR1* (*t*(18) = 3.3, *p* = 0.004) and *CXCR2* (*t*(11.6) = 3.4, *p* = 0.006, Welch's *t* test) that were both significantly increased (~2.5‐fold) in the AUD OFC relative to CONs. We report an approximate 2.1‐fold increase of the CXCL10 receptor *CXCR3* (*t*(13.1) = 4.2, *p* = 0.001, Welch's *t* test) and a 2.4‐fold increase of the CXCL12 receptor *CXCR4* (*t*(18) = 2.7, *p* = 0.014) in the AUD OFC relative to CONs (Figure 5D). Expression of these C‐C motif and C‐X‐C motif chemokines and their corresponding receptors shows significant positive correlations with *HMGB1*, *MYD88*, *TLR2*, and *TLR*s *5*–*9*, but not *TLR3* or *TLR4* (Tables S6 and S7). Together, these data suggest that AUD increases expression of multiple chemokines and their receptors in the human OFC (Figure 5E) in association with induction of HMGB1‐TLR‐MyD88‐NFκB signaling cascades.

### Neuroimmune signaling in the postmortem human OFC correlate with the neurodegeneration marker Fluoro‐Jade B

**FIGURE 6:**
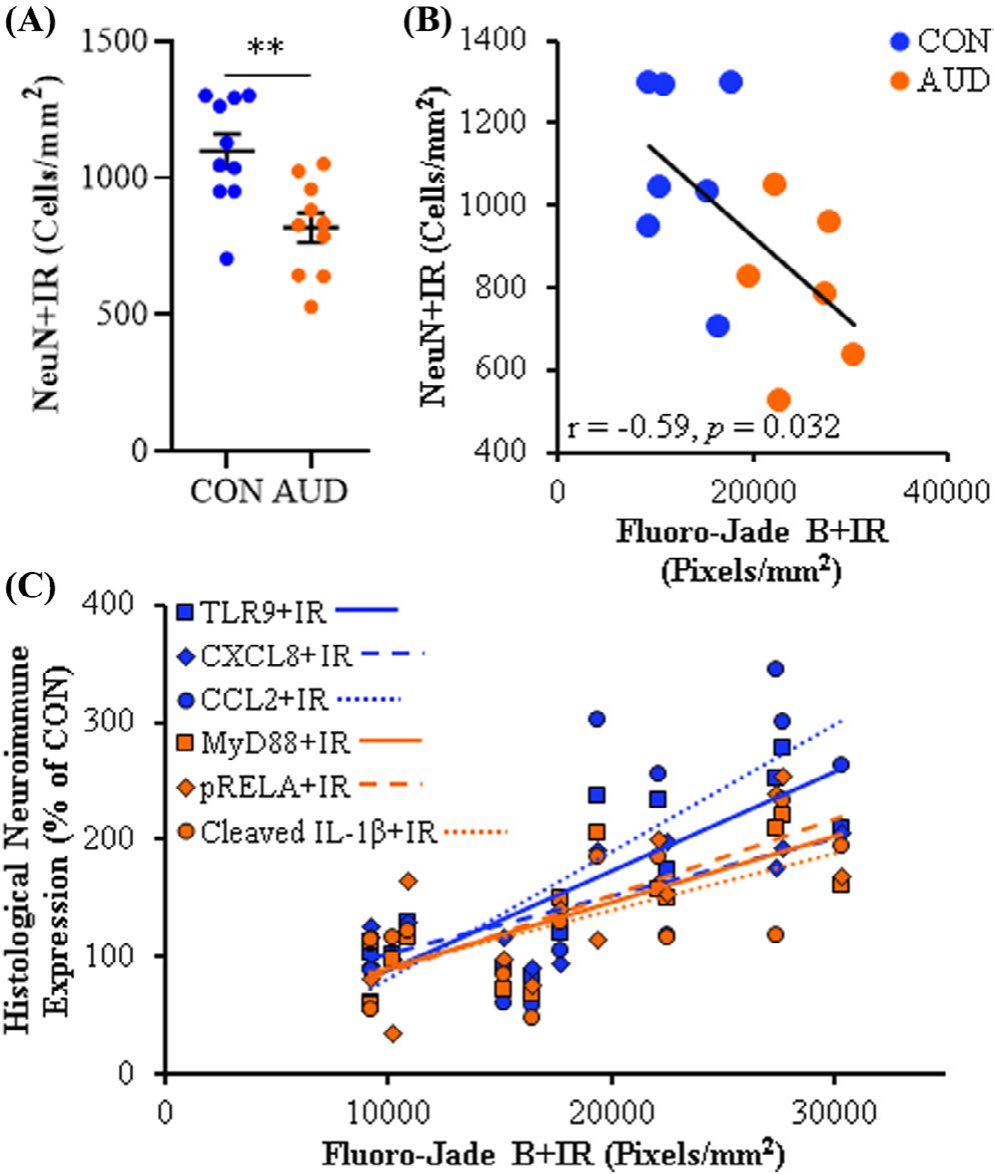
Neurodegeneration in the orbitofrontal cortex (OFC) correlates with expression of neuroimmune markers. (A) Modified unbiased stereological assessment revealed an approximate 26% (±5) reduction of NeuN+IR in the AUD OFC relative to CONs. (B) Across subjects, expression of the neuronal marker NeuN was negatively correlated with the neurodegeneration marker Fluoro‐Jade B (Qin & Crews, 2012b) in the postmortem human OFC (*r* = −0.59, *p* = 0.032). (C) Across subjects, expression of the neurodegeneration marker Fluoro‐Jade B positively correlated with immunohistochemical expression of TLR9 (*r* = 0.84, *p* = 0.0004), CXCL8 (*r* = 0.80, *p* = 0.001), CCL2 (*r* = 0.76, *p* = 0.002), MyD88 (*r* = 0.76, *p* = 0.002), pRELA (*r* = 0.75, *p* = 0.003), and cleaved IL‐1β (*r* = 0.65, *p* = 0.017) in the postmortem human OFC. Data are presented as mean ± SEM. ** *p* < 0.01

Previous preclinical studies have linked chronic alcohol exposure with microglial activation, increases of NADPH oxidase and reactive oxygen species, and increases of cell death markers (e.g., Qin & Crews, 2012b). That earlier study (i.e., Qin & Crews, 2012b) included postmortem human OFC Fluoro‐Jade B histochemistry with some patients overlapping with the current study, thereby allowing correlations of cell death marker expression with proinflammatory TLR signaling cascades. The AUD‐associated increase of Fluoro‐Jade B+IR was accompanied by a 26% (±5) reduction of OFC NeuN+neurons (*t*(18) = −3.4, *p* = 0.0001; Figure 6A), which was negatively correlated (*r* = −0.59, *p* = 0.032) with Fluoro‐Jade B expression (Figure 6B), consistent with neurodegeneration. Across subjects, we observed that multiple neuroimmune signaling molecules significantly correlated with Fluoro‐Jade B+IR (Figure 6C; Table S8). While *TNFA*, IKKβ+IR, *CXCR4*, *CCL8*, and *HMGB1* positively correlated with Fluoro‐Jade B, these correlations were not statistically significant following statistical corrections. Although correlation does not establish causal relationships, the observed correlations are consistent with induction of HMGB1‐TLR‐MyD88‐NFκB signaling cascades in the OFC contributing to neurodegeneration. Further, induction of this signaling cascade across microglia and other cell types likely contributes to neurodegeneration in the postmortem human OFC of individuals with AUD.

Our findings that HMGB1‐TLR‐MyD88‐NFκB cytokine and chemokine neuroimmune signaling cascades correlate with Fluoro‐Jade B+IR prompted comparison of lifetime alcohol consumption (kg) and age of drinking onset with expression of Fluoro‐Jade B+IR and HMGB1‐TLR‐MyD88‐NFκB cytokine and chemokine neuroimmune signaling markers. In this cohort, AUD individuals consumed between 613 and 8052 kg of alcohol, providing a broad range of consumption and showing a progressive increase in neurodegeneration (i.e., Fluoro‐Jade B) and neuroimmune marker expression with increased lifetime alcohol consumption. Fluoro‐Jade B+IR, *TLR4*, MyD88+IR, *NFKB1*, cleaved IL‐1β+IR, CCL2+IR, and CXCL8+IR all showed significant positive correlations with lifetime alcohol consumption (Table S8). Age of drinking onset, which ranged in AUD subjects from 14 to 18 years of age, was negatively correlated with Fluoro‐Jade B+IR and all induced neuroimmune genes that correlated with Fluoro‐Jade B (Table S8), suggesting that a younger age of drinking onset is associated with increased neurodegeneration and neuroimmune induction in the OFC. Our finding that Fluoro‐Jade B+IR and HMGB1‐TLR‐MyD88‐NFκB cytokine and chemokine neuroimmune signaling cascades positively correlate with lifetime alcohol consumption and negatively correlate with age of drinking onset are consistent with early onset, heavy alcohol consumption contributing to increases in TLR proinflammatory signaling cascades and neurodegeneration in the postmortem human OFC.

## DISCUSSION

**FIGURE 7:**
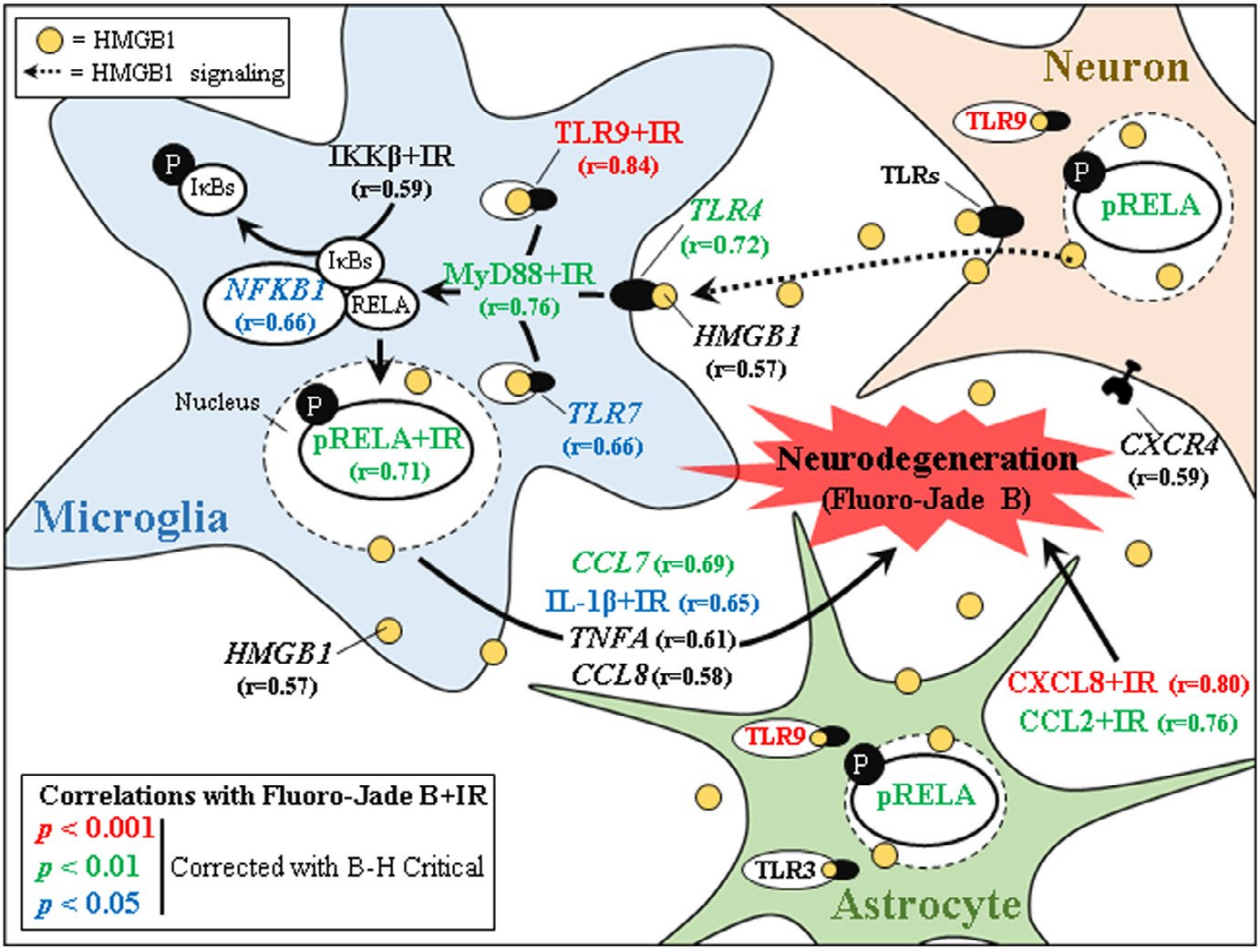
Induction of proinflammatory HMGB1‐TLR‐MyD88‐NFκB neuroimmune signaling across glia and neurons contributes to neurodegeneration in alcohol use disorder (AUD). Simplified schematic of AUD‐induced activation of proinflammatory neuroimmune signaling in the postmortem human orbitofrontal cortex (OFC). Shown are significant Pearson's r correlations of Fluoro‐Jade B+IR data from a previously published study (Qin & Crews, 2012b) with neuroimmune signaling molecules colored for significance in the postmortem human OFC. Red‐labeled correlations indicate statistically significant correlation *p* value < 0.001; green‐labeled correlations indicate statistically significant correlation *p* value < 0.01; blue‐labeled correlations indicate statistically significant correlation *p* value < 0.05 after correction for multiple comparisons using Benjamini‐Hochberg procedure (B‐H Critical) for controlling false positives. Black‐labeled *HMGB1*, IKKβ+IR, *TNFA*, and *CCL8* correlated with Fluoro‐Jade B+IR but did not achieve significance following correction for multiple comparisons using B‐H Critical. Alcohol causes nuclear release of the ubiquitously expressed universal TLR agonist high‐mobility group box 1 (HMGB1) (Crews et al., 2013) that binds to and activates TLRs located on glia and neurons (Crews et al., 2013). Microglia, the resident monocyte‐like cell of the CNS, express all Toll‐like receptors (TLRs) under basal conditions and contribute to initiation of proinflammatory neuroimmune cascades (Block et al., 2007; Soreq et al., 2017). Ligation of HMGB1 to all TLRs, except TLR3, recruits the intracellular TLR coupling protein MyD88, activating downstream activation and nuclear translocation of the proinflammatory mediator nuclear factor kappa B (NFκB) and transcription of proinflammatory cytokines and chemokines. These data implicate induction of HMGB1‐TLR‐MyD88‐NFκB cascades through coordinated glial and neuronal signaling as contributing to neurodegeneration in the postmortem human OFC of individuals with AUD

To our knowledge, this is the first study to report upregulation of a broad number of frontal cortical genes involved in HMGB1‐TLR‐MyD88‐NFκB cytokine and chemokine neuroimmune cascades in the postmortem human OFC of individuals with AUD that we link to neurodegeneration. The RT‐PCR and IHC data presented here, which allows for resolution of AUD‐induced changes in the postmortem human brain that are observed in preclinical models, complement, and extend prefrontal cortex AUD transcriptome studies (Brenner et al., 2020) as well as molecular and histochemical assessments of proinflammatory neuroimmune induction in the AUD brain (Crews et al., 2013; Qin & Crews, 2012b). We report that AUD induces gene expression of *HMGB1* as well as multiple TLRs (i.e., *TLR*s *2*–*9*), but not *TLR1*. Toll‐like receptors are well‐characterized pattern recognition receptors in brain implicated in numerous disease states, including AUD and AD (Coleman & Crews, 2018; Crews et al., 2017; Paudel et al., 2020). HMGB1 is a highly conserved, ubiquitously expressed nuclear protein released from cells in response to injury whereupon it acts as a universal TLR agonist through either direct activation (i.e., TLR4) or indirect activation through formation of heteromers with TLR agonists (e.g., TLR7 and TLR9; Yanai et al., 2009; Andersson & Tracey, 2011; Ivanov et al., 2007). In preclinical studies, alcohol releases HMGB1‐let7 miRNA heteromers in microglial vesicles, activating TLR7‐induced gene expression and neurodegeneration (Coleman et al., 2017; Qin et al., 2021). Studies in bone marrow‐derived DCs find that HMGB1 release with CpG‐DNA, which is a TLR9 ligand, enhances TLR9 proinflammatory gene induction (Ivanov et al., 2007). The ability of TLRs to recognize and respond to endogenous HMGB1 and similar DAMPs results in a sterile inflammatory state wherein innate immune signaling is induced in brain in the absence of invading pathogens (Coleman and Crews, 2018). Indeed, alcohol causes nuclear release and circulation of HMGB1 (Crews et al., 2013). Ligation of HMGB1 to TLRs recruits intracellular adaptor signaling molecules, including MyD88, TRIF, and the viral‐mediated TLR adaptor IRF3. We report that AUD induces MyD88, the downstream NFκB‐activating IKK complex, and the NFκB transcription factors *NFKB1* and *RELA* in the OFC, consistent with TLR coupling to MyD88 activating the proinflammatory nuclear factor NFκB and downstream transcription of proinflammatory signaling molecules (Kawai & Akira, 2007; Pahl, 1999; Schmitz & Baeuerle, 1991). Indeed, we previously reported that AUD increases activated pRELA+IR in the human OFC (Qin et al., 2021), consistent with activation of NFκB. Further, we report that AUD induces proinflammatory cytokines as well as C‐C motif and C‐X‐C motif chemokines and their corresponding receptors, consistent with NFκB transcription of proinflammatory signaling (Pahl, 1999; Schmitz & Baeuerle, 1991). The observed induction of HMGB1‐TLR‐MyD88‐NFκB cytokine and chemokine neuroimmune cascades is accompanied by reductions of NeuN+IR neurons in the AUD OFC that negatively correlate with expression of the neurodegenerative marker Fluoro‐Jade B. Further, expression of Fluoro‐Jade B positively correlates with several of these innate immune genes, implicating AUD‐induced neuroimmune signaling in OFC neurodegeneration in individuals with AUD. Together, these data reveal that AUD induces HMGB1‐TLR‐MyD88‐NFκB cytokine and chemokine expression in the human OFC that likely contributes to neurodegeneration (Figure 7).

Induction of HMGB1‐TLR‐MyD88‐NFκB cytokine and chemokine signaling cascades in the human OFC suggests a concerted neuroimmune response across glia (i.e., microglia and astrocytes) and neurons. Microglia, which are the resident monocyte‐like glia of the brain, express many of these proinflammatory signaling genes, and studies in postmortem human AUD brain (He & Crews, 2008) and preclinical rodent models (Vetreno et al., 2017; Walter et al., 2017) find alcohol exposure increases ramified microglia populations, consistent with microglial activation and induction of proinflammatory cytokines and chemokines. While microglial depletion in mice blocks acute alcohol induction of *TNFA* and *TLR7*, it does not block *HMGB1*, *TLR2*, *TLR3*, *TLR4*, *IL1B*, *IL6*, or *CCL2*, suggesting that these latter genes may be independent of microglia (Walter & Crews, 2017). However, in a recent study, media transfer from ethanol‐treated organotypic hippocampal slice cultures (OHSCs) containing microglial microvesicles to naïve OHSCs induced *TNFA* and other proinflammatory cytokines that was blunted in microglial‐depleted ethanol‐treated media consistent with microglial vesicular signaling contributing to some aspects of alcohol‐induced neuroimmune activation (Crews et al., 2021). Further, while microglia depletion prevents escalations voluntary alcohol intake and concomitant anxiety‐like behavior in alcohol dependent mice, it does not affect voluntary ethanol intake in nondependent mice supporting a role for microglia in alcohol dependence (Warden et al., 2021; Warden et al., 2020). The observation that numerous proinflammatory neuroimmune genes are unaltered following microglial depletion *in vivo* suggests that astrocytes and neurons express higher levels of neuroimmune mediators than previously known. Indeed, astrocytes and neurons are emerging as important contributors to neuroimmune responses (Erickson et al., 2021; Lawrimore et al., 2019; Lawrimore & Crews, 2017; Moffat & Ron, 2021) and a recent cell type‐specific transcriptome study in the postmortem human brain revealed unique neuroimmune gene signatures across glia and neurons with robust changes in astrocytes (Brenner et al., 2020; Erickson et al., 2019a; Soreq et al., 2017). While *HMGB1* is enriched across all cell types, *TLR2*, *TLR7*, and *TLR9* are particularly enriched in microglia at baseline, whereas *TLR3* is enriched in astrocytes and the remaining TLRs are modestly expressed across glia and neurons (Soreq et al., 2017). However, alcohol exposure can cause induction in other cell types. In the present study, we report TLR9 co‐expression with microglia as well as astrocytes and neurons, consistent with previous studies reporting colocalization of TLR2, TLR3, and TLR4 with neurons in the postmortem human brain (Casula et al., 2011; Crews et al., 2013; Maroso et al., 2010; Zurolo et al., 2011). All TLRs, except TLR3, signal through the TLR adaptor protein MyD88, which is expressed across cell types but is particularly enriched in microglia and astrocytes (Brenner et al., 2020; Soreq et al., 2017). Alcohol releases HMGB1 from neurons and microglia (Crews et al., 2013; Lawrimore & Crews, 2017), and we find *HMGB1* is positively correlated with all induced TLRs except *TLR3*. While correlation does not establish causal relationships, correlation of *HMGB1* with multiple TLRs and *MYD88* in light of the aforementioned literature is consistent with HMGB1 acting as a global TLR agonist across cell types that signal through MyD88. Knockout of MyD88 or the associated adaptor protein TIRAP in cultured macrophages blocks assessed TLR1, TLR2, TLR4, TLR6, TLR7, and TLR9 ligand activation and blunts downstream NFκB signaling and induction of proinflammatory cytokines IL6 and TNFα, but not IL‐1β (Horng et al., 2002; Kawai et al., 2001). Members of the NFκB‐activating IKK complex are expressed in neurons, astrocytes, and microglia in the human brain, whereas *NFKB1* and *RELA* are similarly expressed across cell types, but particularly enriched in microglia (Soreq et al., 2017). In the human OFC, activated pRELA colocalizes with glial and neuronal markers, and the majority of induced NFκB signaling and family genes correlate with *HMGB1*, *TLR*s, and *MYD88*, further supporting induction of proinflammatory HMGB1‐TLR‐MyD88‐NFκB signaling across glia and neurons. We report CCL2 and CXCL8 are co‐expressed by microglia, astrocytes, and neurons in the human OFC, with the majority of induced cytokine and chemokine genes correlating with expression of *HMGB1*, *TLR*s, and *MYD88*. Somewhat surprising was our finding that *IL1B* and *TNFA* did not correlate with TLR expression, suggestive of different signaling mechanisms (Horng et al., 2002; Qin et al., 2007). Taken together, these data suggest that induction of HMGB1‐TLR‐MyD88‐NFκB cytokine and chemokine signaling in the human OFC involves a coordinated cascade of neuroimmune signaling involving microglia as well as astrocytes and neurons.

Proinflammatory neuroimmune induction and neurodegeneration are hallmark features of neurodegenerative disorders and addiction (Crews et al., 2017; Kempuraj et al., 2016; Kohno et al., 2019). Human neuroimaging studies report decreased OFC volume and brain connectivity (Moorman, 2018) in AUD individuals, and postmortem histological studies find decreased glial and neuronal packing density, the latter of which correlates with duration of alcohol dependence, in the OFC of individuals with AUD (Miguel‐Hidalgo et al., 2006). Likewise, we report reductions of the neuronal marker NeuN that negatively correlate with expression of the neurodegeneration marker Fluoro‐Jade B. We find increased markers of cell death in human AUD primarily reflect an apoptotic cell death process (Liu et al., 2020) but have also observed direct evidence of neurodegeneration through colocalization of NeuN with Fluoro‐Jade B in the postmortem human OFC of individuals with AUD (Qin & Crews, 2012b). In preclinical studies, alcohol treatment similarly increases cortical apoptotic cell death and caspase‐3 activity in the rat neocortex, effects that are blocked by treatment with the anti‐inflammatory drug indomethacin (Pascual et al., 2007). Similarly, TLR4 knockout in mice blocks alcohol‐induced upregulation of caspase‐3 (Alfonso‐Loeches et al., 2010), implicating neuroimmune induction through TLRs in alcohol‐induced neurodegeneration. It is interesting to note that microglial depletion does not block genes encoding death receptor or death receptor ligands, suggesting that apoptotic signaling occurs independent of microglia (Walter & Crews, 2017). The AUD‐induced increases of HMGB1‐TLR‐MyD88‐NFκB cytokine and chemokine neuroimmune signaling are likely the result of both alcohol drinking and alcohol‐induced neurodegeneration. These findings are consistent with chronic alcohol exposure progressively inducing persistent HMGB1‐TLR‐MyD88‐NFκB cytokine and chemokine neuroimmune signaling that may contribute to the modest and diffuse neurodegeneration associated with AUD relative to AD and other neurodegenerative disorders that may sensitize the brain to age‐related neurodegeneration and disease.

While neurodegeneration is an important endpoint in this study and neuroimmune signaling molecules are expressed by glia and neurons, accumulating evidence implicates neuroimmune induction in regulating brain function. Neuroimmune signaling is thought to contribute to increased alcohol drinking and drug addiction as knockout of many key neuroimmune genes, including *TLR4*, *IL6*, and *CCL2*, decreases alcohol consumption in mice (Blednov et al., 2005; Blednov et al., 2012). Interestingly, we report that an earlier age of drinking onset negatively correlates with HMGB1‐TLR‐MyD88‐NF‐κB cytokine and chemokine signaling, likely contributing to the increased risk for adult AUD development in individuals that start drinking at a younger age (Dawson et al., 2008; Grant, 1998). Neuroimmune induction also appears to contribute to the withdrawal and negative affect associated with AUD as brain levels of several proinflammatory signaling molecules, including TNFα and CCL2, are increased during alcohol withdrawal in mice (Qin et al., 2008), and ventricular injection of TNFα or CCL2 potentiates alcohol‐induced withdrawal‐associated negative affect in rats (Breese et al., 2008). Further, in humans with AUD, plasma levels of several circulating cytokines (e.g., TNFα, IL‐1β, IL‐6) and chemokines (e.g., CXCL8) correlate with alcohol craving (Heberlein et al., 2014; Leclercq et al., 2014). Inhibition of amygdalar IL‐1β reduces binge‐like alcohol drinking (Marshall et al., 2016). Similarly, inhibition of IL‐1β in the ventral tegmental area prevents cocaine‐induced dopamine release in the nucleus accumbens (Northcutt et al., 2015) whereas neuronal NFκB is essential for amplification of cocaine addiction (Russo et al., 2009), further implicating neuroimmune signaling in addictive behaviors. Recently, AUD and alcohol abuse in humans has emerged as a potential etiological factor contributing to the onset of dementia later in life (Kamal et al., 2020). Thus, our finding of AUD‐induced increases of HMGB1‐TLR‐MyD88‐NFκB cytokine and chemokine neuroimmune signaling suggests this signaling cascade should be considered in studies linking innate immune signaling to mood, cognition, and addiction.

In conclusion, we report induction of the universal endogenous TLR agonist HMGB1 in the postmortem human OFC of individuals with AUD that parallels increases of all TLRs (i.e., TLR2‐TLR9) except TLR1. Induction of HMGB1‐TLR signaling is accompanied by increased expression of the TLR adaptor protein MyD88, activation of the proinflammatory nuclear transcription factor NFκB, and downstream induction of proinflammatory cytokines, chemokines, and their corresponding receptors. Expression of several of these proinflammatory signaling markers is observed in glia and neurons. Induction of the HMGB1‐TLR‐MyD88‐NFκB proinflammatory signaling pathways correlates with neurodegeneration (i.e., Fluoro‐Jade B) as well as lifetime alcohol consumption and age of drinking onset. These data implicate induction of HMGB1‐TLR‐MyD88‐NFκB cascades through coordinated glial and neuronal signaling as contributing to neurodegeneration in the postmortem human OFC of individuals with AUD.

## CONFLICTS OF INTEREST

None.

## Supporting information

Fig S1

Supplementary Material

Table S1

Table S2

Table S3

Table S4

Table S5

Table S6

Table S7

Table S8
