# Supplementary material for: Increased Toll‐like Receptor‐MyD88‐NFκB‐Proinflammatory neuroimmune signaling in the orbitofrontal cortex of humans with alcohol use disorder
Source: Alcohol Clin Exp Res. 2021 Aug 20;45(9):1747–61. doi: 10.1111/acer.14669 (PMC8526379; doi:10.1111/acer.14669)
Supplement: Supplementary file 2 — Supplementary Material [file ACER-45-1747-s004.docx]

**Supplementary material**

**Supplementary Fig. 1. Representative fluorescent immunohistochemical photomicrographs of neuroimmune signaling marker colocalization with glial markers in the post-mortem human orbitofrontal cortex (OFC).** (A) High-magnification photomicrographs of Toll-like receptor 9 (TLR9; green) colocalization with the microglial marker Iba1 (middle; red) in the post-mortem human OFC of an individual with alcohol use disorder (AUD). White arrows indicate TLR9+IR cells that colocalize with Iba1 (yellow). (B) High-magnification photomicrographs of TLR9 (green) colocalization with the astroglial marker glial fibrillary acidic protein (GFAP; red) in the post-mortem human OFC of an individual with AUD. White arrows indicate TLR9+IR cells that colocalize with GFAP (yellow). (C) High-magnification photomicrographs of phosphorylated RELA (pRELA; green) colocalization with Iba1 (red) in the post-mortem human OFC of an individual with AUD. White arrows indicate pRELA+IR cells that colocalize with Iba1 (yellow). (D) High-magnification photomicrographs of pRELA (green) colocalization with GFAP (red) in the post-mortem human OFC of an individual with AUD. White arrows indicate representative pRELA+IR cells that colocalize with GFAP (yellow). (E) High-magnification photomicrographs of CCL2 (green) colocalization with Iba1 (red) in the post-mortem human OFC of an individual with AUD. White arrows indicate CCL2+IR cells that colocalize with Iba1 (yellow). (F) High-magnification photomicrographs of CCL2 (green) colocalization with GFAP (red) in the post-mortem human OFC of an individual with AUD. White arrows indicate CCL2+IR cells that colocalize with GFAP (yellow). Scale bar = 20 μm. (G) High-magnification photomicrographs of CXCL8 (green) colocalization with Iba1 (red) in the post-mortem human OFC of an individual with AUD. White arrows indicate CXCL8+IR cells that colocalize with Iba1 (yellow). (H) High-magnification photomicrographs of CXCL8 (green) colocalization with GFAP (red) in the post-mortem human OFC of an individual with AUD. White arrows indicate CXCL8+IR cells that colocalize with GFAP (yellow). Scale bar = 20 μm.
