# Supplementary material for: Increased Toll‐like Receptor‐MyD88‐NFκB‐Proinflammatory neuroimmune signaling in the orbitofrontal cortex of humans with alcohol use disorder
Source: Alcohol Clin Exp Res. 2021 Aug 20;45(9):1747–61. doi: 10.1111/acer.14669 (PMC8526379; doi:10.1111/acer.14669)
Supplement: Supplementary file 3 — Table S1 [file ACER-45-1747-s002.docx]

| **Supplementary Table 1.** List of primers for human reverse transcription PCR (RTPCR). | | |
| --- | --- | --- |
| Primer | Forward | Reverse |
| *TLR1* | 5'-GGCATATTGGGCACCCCTAC-3' | 5'-AGGAACGTGGATGAGACCG-3' |
| *TLR2* | 5'-CCTGCCTGCTAGTGATAGGTG-3' | 5'-GAATGGAGGAATGAGCAATCAAGC-3' |
| *TLR3* | 5'-GTGCCGTCTATTTGCCACAC-3' | 5'-AGTTGGCGGCTGGTAATCTT-3' |
| *TLR4* | 5'-TACAAAATCCCCGACAACCTC-3' | 5'-AGCCACCAGCTTCTGTAAACT-3' |
| *TLR5* | 5'-GGGACTAAGCCTCAACTCCA-3' | 5'-GGAAACCCCAGAGAACGAGT-3' |
| *TLR6* | 5'-GCGGACTTATTTGCAGTGGC-3' | 5'-CTCACCGCCTAGCTCAGTTC-3' |
| *TLR7* | 5'-GATAACAATGTCACAGCCG-3' | 5'-GTTCCTGGAGTTTGTTGATG-3' |
| *TLR8* | 5'-ATGTTCCTTCAGTCGTCAAT-3' | 5'-TTGCTGCACTCTGCAATAAGC-3' |
| *TLR9* | 5'-CCGTGACAATTACCTGGCCT-3' | 5'-GAGGGTTGGCGCTTACATCT-3' |
| *HMGB1* | 5'-GGAGATCCTAAGAAGCCGAGA-3' | 5'-CATGGTCTTCCACCTCTCTGA-3' |
| *MYD88* | 5'-GAGTGGAAGAGGTTTGGAAGC-3' | 5'-GCCCTGCTTAGGACTGAACC-3' |
| *TICAM1* | 5'-CTTCAGGATGAGGCCCGAAA-3' | 5'-GGGGACTGGCTGATTTCCAA-3' |
| *IRF3* | 5'-CTGGCGGAATTGAGGGAGTG-3' | 5'-CAGGAAACCTCCTCTTCCCAT-3' |
| *NFKB1* | 5'-GTCACCTCCTTTGAACAGCTT-3' | 5'-GGCTGGTTGACTAACTGGCT-3' |
| *NFKB2* | 5'-ACCTCAAACTCCAGGTAGGC-3' | 5'-CTGTATAGGGCAGAGTGACCA-3' |
| *RELA* | 5'-CATTGGTGGTAGAGAGCTGGG-3' | 5'-CGCTGGCTAGTCCCTCTTTG-3' |
| *RELB* | 5'-CCTCATATCGGGACCAGCAG-3' | 5'-GTCACGGGCTCGACAATCT-3' |
| *REL* | 5'-AGTTGCGGAGACCTTCTGAC-3' | 5'-AATTGAACCGAGGAGACCAG-3' |
| *IKBKB* | 5'-GCAGAAGAGTGAGGTGGACATT-3' | 5'-GTGTAATGCCGCTGTGCCTT-3' |
| *IKBKG* | 5'-CCACTTGCCTCGGGCTAATC-3' | 5'-TACCATCTACGCCATCGCCC-3' |
| *NFKBIA* | 5'-GCATCGTGGAGCTTTTGGTG-3' | 5'-CCCTTTGCGCTCATAACGTC-3' |
| *NFKBIB* | 5'-GCCTCTCTGCTTTCTCTTTCCACTT-3' | 5'-CCAGGGTCCATTTGGGACATAGT-3' |
| *NFKBIE* | 5'-GGAGCCTGGGGATAAGGGTA-3' | 5'-CTGTGCTGGTTTATTGCCCC-3' |
| *IL1B* | 5'-TGAAGCTGATGGCCCTAAACA-3' | 5'-GCCTGAAGCCCTTGCTGTAGT-3' |
| *IL1R1* | 5'-CCTGTCTTATGGCGTTGCAGGC-3' | 5'-AGTGCCCTGGGCTGCTATTGAC-3' |
| *IL1RN* | 5'-ATGTTGGGTGCAAAGTTCCC-3' | 5'-GTCAAGGCCAAGGACATAGTCA-3' |
| *IL6* | 5'-CCCCCAGGAGAAGATTCCAAAG-3' | 5'-TTCTGCCAGTGCCTCTTTGCTG-3' |
| *IL6R* | 5'-CCAGTAGTGTCGGGAGCAAG-3' | 5'-GTCCTTGACCATCCATGTTGTG-3' |
| *TNFA* | 5'-CCCAGGCAGTCAGATCATCTT-3' | 5'-TCTCAGCTCCACGCCATT-3' |
| *TNFRSF1A* | 5'-CTGGACAAGCACATAGCAAGC-3' | 5'-GTCACTTGGCGTGATGGTGA-3' |
| *ADAM17* | 5'-GTGACATGAATGGCAAATGTGAGA-3' | 5'-ACAATGGACAAGAATGCTGAAAGG-3' |
| *CCL2* | 5'-GATGCAATCAATGCCCCAGTC-3' | 5'-TCCTTGGCCACAATGGTCTTG-3' |
| *CCL3* | 5'-GCTGACTACTTTGAGACGAGC-3' | 5'-CCAGTCCATAGAAGAGGTAGC-3' |
| *CCL4* | 5'-CAGCGCTCTCAGCACCAATGG-3' | 5'-GATCAGCACAGACTTGCTTGCTTC-3' |
| *CCL5* | 5'-GATTGGGATGACAGGGCTGAA-3' | 5'-GATGCTGTGGCAGGCAGTAA-3' |
| *CCL7* | 5'-GCCTCTGCAGCACTTCTGTG-3' | 5'-CACTTCTGTGTGGGGTCAGC-3' |
| *CCL8* | 5'-CCCAGGTGCAGTGTGACAT-3' | 5'-CATCACCAGGATTTTGCATTT-3' |
| *CCL13* | 5'-GGCCTTGAGCAAGTAGGTTG-3' | 5'-CATGTCCATGACTCCCACAG-3' |
| *CCL19* | 5'-GACCTCAGCCAAGATGAAGC-3' | 5'-GGTCCTTCCTTCTGGTCCTC-3' |
| *CCR1* | 5'-CCCAGTGATCTACGCCTTCG-3' | 5'-CCCCAGGCCACCATTACATT-3' |
| *CCR2* | 5'-TTGCCCCACTCCAAAAACCA-3' | 5'-CCTTCCTGCCTGGTAACGTA-3' |
| *CXCL8* | 5'-ATAAAAAGCCACCGGAGCACT-3' | 5'-ACAGTGAGATGGTTCCTTCCG-3' |
| *CXCL10* | 5'-GCTGTACCTGCATCAGCATT-3' | 5'-GCTCCCCTCTGGTTTTAAGG-3' |
| *CXCL12* | 5'-GAGGGTTGTGTAATCCTGGC-3' | 5'-GGAGCACCTTTCCCTGCTAA-3' |
| *CXCR1* | 5'-CGTCATCGTGTTACCTCCTACA-3' | 5'-GCCAGATCACCTTCCACACA-3' |
| *CXCR2* | 5'-AATCCCCAGCACTCATCCCAG-3' | 5'-GGGCTTTTCACCTGTAGGACAC-3' |
| *CXCR3* | 5'-GGCATGTTCGTGCATCATTACA-3' | 5'-GCAGGGCTCTCGTGTTCAT-3' |
| *CXCR4* | 5'-CCAGCCAGCACCTATTTGTATG-3' | 5'-GGCTTTGCCCCCTTGAAAGT-3' |
| *ACTB* | 5'-GCATGGGTCAGAAGGATTCCT-3' | 5'-TCGTCCCAGTTGGTGACGAT-3' |
