# Supplementary material for: Increased Toll‐like Receptor‐MyD88‐NFκB‐Proinflammatory neuroimmune signaling in the orbitofrontal cortex of humans with alcohol use disorder
Source: Alcohol Clin Exp Res. 2021 Aug 20;45(9):1747–61. doi: 10.1111/acer.14669 (PMC8526379; doi:10.1111/acer.14669)
Supplement: Supplementary file 4 — Table S2 [file ACER-45-1747-s006.docx]

| **Supplementary Table 2.** List of primary antibodies used for immunohistochemistry in the post-mortem human orbitofrontal cortex. | | | | | |
| --- | --- | --- | --- | --- | --- |
| Antibody | Isotype | Source/ Purification | Dilution | Company, Catalog Number | Validation |
| TLR9 | Rabbit IgG | Polyclonal | 1:100 | Invitrogen, #PA5-27258 | WB, IHC (Invitrogen) |
| MyD88 | Rabbit IgG | Polyclonal | 1:50 | Abcam Inc., #ab2064 | WB (Abcam Inc.) |
| NFκB p65 phospho S536 | Rabbit IgG | Polyclonal | 1:150 | Abcam Inc., #ab86299 | WB, IHC^1^ |
| IKKβ | Rabbit IgG | Polyclonal | 1:70 | Novus Biologicals, #NB600-477 | WB^2^ |
| Cleaved IL-1β | Rabbit IgG | Polyclonal | 1:100 | Abcam Inc., #ab2105 | WB^3^ |
| CCL2 [MCP-1] | Mouse IgG | Monoclonal | 1:150 | Millipore, #MABN712 | WB (Millipore) |
| CXCL8 [807] | Mouse IgG | Monoclonal | 1:500 | Abcam Inc., #ab18672 | WB, IHC^4^ |
| Validation information includes references as well as methods used for validation and source. ^1^Xiao, F., Zheng, R., Yang, D., Cao, K. et al. (2017). Sex-dependent aortic valve pathology in patients with rheumatic heart disease. PLoS One, 12, e0180230. ^2^Liu, Y., Liu, X., Hao, W., Decker, Y. et al. (2014). IKKβ deficiency in myeloid cells ameliorates Alzheimer's disease-related symptoms and pathology. J. Neuroscience, 34, 12982-12999. ^3^Gui, W., Wei, X., Mai, C., Murugan, M. (2016). Interleukin-1β overproduction is a common cause for neuropathic pain, memory deficit, and depression following peripheral nerve injury in rodents. Mol Pain, 12, 1-15. ^4^Levina, V., Su, Y., Nolen, B., Liu, X. et al. (2008). Chemotherapeutic drugs and human tumor cells cytokine network. Int J Cancer, 123, 2031-2040. | | | | | |
