# Supplementary material for: Increased Toll‐like Receptor‐MyD88‐NFκB‐Proinflammatory neuroimmune signaling in the orbitofrontal cortex of humans with alcohol use disorder
Source: Alcohol Clin Exp Res. 2021 Aug 20;45(9):1747–61. doi: 10.1111/acer.14669 (PMC8526379; doi:10.1111/acer.14669)
Supplement: Supplementary file 5 — Table S3 [file ACER-45-1747-s008.docx]

| **Supplementary Table 3.** Correlations of Toll-like receptor (*TLR*), high mobility group box 1 (*HMGB1*), *MYD88*, and *TICAM1* genes in the post-mortem human orbitofrontal cortex (OFC) of age-matched moderate drinking control (CON) and alcohol use disorder (AUD) individuals. | | | | | | | | | | |
| --- | --- | --- | --- | --- | --- | --- | --- | --- | --- | --- |
|  | *TLR3* | *TLR4* | *TLR5* | *TLR6* | *TLR7* | *TLR8* | *TLR9* | *HMGB1* | *MYD88* | *TICAM1* |
| *TLR2* | 0.10 | 0.24 | 0.91 ** | 0.90 ** | 0.45 * | 0.76 ** | 0.77 ** | 0.69 ** | 0.71 ** | -0.54 * |
| *TLR3* |  | 0.23 | 0.13 | 0.29 | 0.71 ** | 0.13 | 0.27 | 0.43 | 0.36 | -0.17 |
| *TLR4* |  |  | 0.11 | 0.27 | 0.40 | 0.44 | 0.16 | 0.49 * | 0.38 | -0.31 |
| *TLR5* |  |  |  | 0.86 ** | 0.48 * | 0.83 ** | 0.90 ** | 0.79 ** | 0.77 ** | -0.55 * |
| *TLR6* |  |  |  |  | 0.59 ** | 0.77 ** | 0.77 ** | 0.72 ** | 0.72 ** | -0.50 * |
| *TLR7* |  |  |  |  |  | 0.68 ** | 0.66 ** | 0.67 ** | 0.77 ** | -0.37 |
| *TLR8* |  |  |  |  |  |  | 0.91 ** | 0.80 ** | 0.89 ** | -0.41 |
| *TLR9* |  |  |  |  |  |  |  | 0.82 ** | 0.87 ** | -0.44 |
| *HMGB1* |  |  |  |  |  |  |  |  | 0.82 ** | -0.51 * |
| *MyD88* |  |  |  |  |  |  |  |  |  | -0.38 |
| Pearson's r correlations assessed the association of TLR-associated genes in post-mortem human OFC tissue samples from CON and AUD subjects. Pearson's r correlation coefficients were used with two-tailed significance. * *p* < 0.05, ** *p* < 0.01. | | | | | | | | | | |
