# Supplementary material for: Increased Toll‐like Receptor‐MyD88‐NFκB‐Proinflammatory neuroimmune signaling in the orbitofrontal cortex of humans with alcohol use disorder
Source: Alcohol Clin Exp Res. 2021 Aug 20;45(9):1747–61. doi: 10.1111/acer.14669 (PMC8526379; doi:10.1111/acer.14669)
Supplement: Supplementary file 6 — Table S4 [file ACER-45-1747-s007.docx]

| **Supplementary Table 4.** Correlations of Toll-like receptor (*TLR*), high mobility group box 1 *(HMGB1),* and *MYD88* with NFκB family and signaling genes in the post-mortem human orbitofrontal cortex (OFC) of age-matched moderate drinking control (CON) and alcohol use disorder (AUD) individuals. | | | | | | |
| --- | --- | --- | --- | --- | --- | --- |
|  | *NFKB1* | *RELA* | *IKBKB* | *IKBKG* | *NFKBIB* | *NFKBIE* |
| *TLR2* | 0.59 ** | 0.81 ** | 0.81 ** | 0.66 ** | 0.67 ** | 0.81 ** |
| *TLR3* | 0.66 ** | 0.17 | 0.24 | 0.20 | 0.35 | 0.29 |
| *TLR4* | 0.46 | 0.13 | 0.27 | 0.06 | 0.08 | 0.19 |
| *TLR5* | 0.59 ** | 0.86 ** | 0.91 ** | 0.84 ** | 0.81 ** | 0.91 ** |
| *TLR6* | 0.75 ** | 0.79 ** | 0.79 ** | 0.62 ** | 0.72 ** | 0.78 ** |
| *TLR7* | 0.93 ** | 0.41 | 0.65 * | 0.52 * | 0.63 ** | 0.63 ** |
| *TLR8* | 0.75 ** | 0.71 ** | 0.94 ** | 0.84 ** | 0.87 ** | 0.89 ** |
| *TLR9* | 0.70 ** | 0.84 ** | 0.96 ** | 0.92 ** | 0.90 ** | 0.96 ** |
| *HMGB1* | 0.78** | 0.62 ** | 0.83 ** | 0.80** | 0.82 ** | 0.84 ** |
| *MYD88* | 0.80 ** | 0.64 ** | 0.90 ** | 0.84 ** | 0.88 ** | 0.92 ** |
| Pearson's r correlations assessed the association of TLR-associated genes with NFκB family and signaling genes in post-mortem human OFC tissue samples from CON and AUD individuals Pearson's r correlation coefficients were used with two-tailed significance. * *p* < 0.05, ** *p* < 0.01. | | | | | | |
