# Supplementary material for: Increased Toll‐like Receptor‐MyD88‐NFκB‐Proinflammatory neuroimmune signaling in the orbitofrontal cortex of humans with alcohol use disorder
Source: Alcohol Clin Exp Res. 2021 Aug 20;45(9):1747–61. doi: 10.1111/acer.14669 (PMC8526379; doi:10.1111/acer.14669)
Supplement: Supplementary file 7 — Table S5 [file ACER-45-1747-s009.docx]

| **Supplementary Table 5.** Correlations of Toll-like receptor (*TLR*), high mobility group box 1 *(HMGB1),* and *MYD88* with cytokine genes in the post-mortem human orbitofrontal cortex (OFC) of age-matched moderate drinking control (CON) and alcohol use disorder (AUD) individuals. | | | | | | |
| --- | --- | --- | --- | --- | --- | --- |
|  | *IL1B* | *IL1R* | *IL1RN* | *IL6* | *TNFA* | *TNFRSF1A* |
| *TLR2* | 0.14 | 0.75 ** | 0.79 ** | 0.77 ** | 0.47 * | 0.82 ** |
| *TLR3* | 0.27 | 0.33 | 0.46 * | 0.28 | 0.23 | 0.42 |
| *TLR4* | 0.27 | 0.19 | 0.40 | 0.30 | 0.14 | 0.56 * |
| *TLR5* | 0.38 | 0.83 ** | 0.86 ** | 0.87 ** | 0.38 | 0.85 ** |
| *TLR6* | 0.11 | 0.76 ** | 0.82 ** | 0.80 ** | 0.33 | 0.85 ** |
| *TLR7* | 0.39 | 0.67 ** | 0.79 ** | 0.65 ** | 0.27 | 0.68 ** |
| *TLR8* | 0.29 | 0.82 ** | 0.91 ** | 0.87 ** | 0.10 | 0.83 ** |
| *TLR9* | 0.46 | 0.91 ** | 0.94 ** | 0.90 ** | 0.23 | 0.86 ** |
| *HMGB1* | 0.38 | 0.74 ** | 0.92 ** | 0.91 ** | 0.17 | 0.81 ** |
| *MYD88* | 0.25 | 0.89 ** | 0.91 ** | 0.80 ** | 0.18 | 0.91 ** |
| Pearson's r correlations assessed the association of TLR-associated genes with cytokine genes in post-mortem human OFC tissue samples from CON and AUD subjects. Pearson's r correlation coefficients were used with two-tailed significance. * *p* < 0.05, ** *p* < 0.01. | | | | | | |
