# Supplementary material for: Increased Toll‐like Receptor‐MyD88‐NFκB‐Proinflammatory neuroimmune signaling in the orbitofrontal cortex of humans with alcohol use disorder
Source: Alcohol Clin Exp Res. 2021 Aug 20;45(9):1747–61. doi: 10.1111/acer.14669 (PMC8526379; doi:10.1111/acer.14669)
Supplement: Supplementary file 8 — Table S6 [file ACER-45-1747-s003.docx]

| **Supplementary Table 6.** Correlations of Toll-like receptor (TLR), high mobility group box 1 (HMGB1), and MyD88 with C-C motif chemokine and receptor genes in the post-mortem human orbitofrontal cortex (OFC) of age-matched moderate drinking control (CON) and alcohol use disorder (AUD) individuals. | | | | | | |
| --- | --- | --- | --- | --- | --- | --- |
|  | *CCL8* | *CCL7* | *CCL13* | *CCL5* | *CCR1* | *CCR2* |
| *TLR2* | 0.79 ** | 0.50 * | 0.77 ** | 0.78 ** | 0.52 * | 0.80 ** |
| *TLR3* | 0.25 | 0.39 | 0.35 | 0.38 | 0.43 | 0.47 * |
| *TLR4* | 0.40 | 0.50 * | 0.33 | 0.35 | 0.07 | 0.27 |
| *TLR5* | 0.86 ** | 0.36 | 0.87 ** | 0.87 ** | 0.64 ** | 0.87 ** |
| *TLR6* | 0.79 ** | 0.49 * | 0.78 ** | 0.80 ** | 0.46 * | 0.86 ** |
| *TLR7* | 0.67 ** | 0.42 | 0.70 ** | 0.68 ** | 0.48 * | 0.68 ** |
| *TLR8* | 0.92 ** | 0.24 | 0.90 ** | 0.90 ** | 0.41 | 0.83 ** |
| *TLR9* | 0.90 ** | 0.27 | 0.94 ** | 0.93 ** | 0.57 ** | 0.89 ** |
| *HMGB1* | 0.86 ** | 0.29 | 0.88 ** | 0.87 ** | 0.54 * | 0.92 ** |
| *MYD88* | 0.95 ** | 0.26 | 0.94 ** | 0.91 ** | 0.54 * | 0.82 ** |
| Pearson's r correlations assessed the association of TLR-associated genes with C-C motif chemokine and receptor genes in post-mortem human OFC tissue samples from CON and AUD subjects. Pearson's r correlation coefficients were used with two-tailed significance. * *p* < 0.05, ** *p* < 0.01. | | | | | | |
