# Supplementary material for: Increased Toll‐like Receptor‐MyD88‐NFκB‐Proinflammatory neuroimmune signaling in the orbitofrontal cortex of humans with alcohol use disorder
Source: Alcohol Clin Exp Res. 2021 Aug 20;45(9):1747–61. doi: 10.1111/acer.14669 (PMC8526379; doi:10.1111/acer.14669)
Supplement: Supplementary file 9 — Table S7 [file ACER-45-1747-s005.docx]

| **Supplementary Table 7.** Correlations of Toll-like receptor (TLR), high mobility group box 1 (HMGB1), and MyD88 with C-X-C motif chemokine and receptor genes in the post-mortem human orbitofrontal cortex (OFC) of age-matched moderate drinking control (CON) and alcohol use disorder (AUD) individuals. | | | | | | |
| --- | --- | --- | --- | --- | --- | --- |
|  | *CXCL8* | *CXCL12* | *CXCR1* | *CXCR2* | *CXCR3* | *CXCR4* |
| *TLR2* | 0.53 * | 0.84 ** | 0.73 ** | 0.75 ** | 0.68 ** | 0.88 ** |
| *TLR3* | 0.33 | 0.11 | 0.48 * | 0.47 * | 0.46 * | 0.21 |
| *TLR4* | 0.02 | 0.36 | 0.29 | 0.32 | 0.09 | 0.44 |
| *TLR5* | 0.46 * | 0.93 ** | 0.84 ** | 0.82 ** | 0.79 ** | 0.87 ** |
| *TLR6* | 0.61 ** | 0.77 ** | 0.80 ** | 0.76 ** | 0.66 ** | 0.89 ** |
| *TLR7* | 0.36 | 0.54 * | 0.79 ** | 0.73 ** | 0.67 ** | 0.68 ** |
| *TLR8* | 0.33 | 0.94 ** | 0.89 ** | 0.86 ** | 0.70 ** | 0.92 ** |
| *TLR9* | 0.41 | 0.94 ** | 0.95 ** | 0.91 ** | 0.86 ** | 0.86 ** |
| *HMGB1* | 0.19 | 0.76 ** | 0.90 ** | 0.85 ** | 0.72 ** | 0.84 * |
| *MYD88* | 0.28 | 0.88 ** | 0.91 ** | 0.95 ** | 0.70 ** | 0.88 ** |
| Pearson's r correlations assessed the association of TLR-associated genes with C-X-C chemokine and receptor genes in post-mortem human OFC tissue samples from CON and AUD subjects. Pearson's r correlation coefficients were used with two-tailed significance. * *p* < 0.05, ** *p* < 0.01. | | | | | | |
