# Supplementary material for: Increased Toll‐like Receptor‐MyD88‐NFκB‐Proinflammatory neuroimmune signaling in the orbitofrontal cortex of humans with alcohol use disorder
Source: Alcohol Clin Exp Res. 2021 Aug 20;45(9):1747–61. doi: 10.1111/acer.14669 (PMC8526379; doi:10.1111/acer.14669)
Supplement: Supplementary file 10 — Table S8 [file ACER-45-1747-s010.docx]

| **Supplementary Table 8.** Correlations of induced neuroimmune genes with Fluoro-Jade B immunoreactivity (+IR), age of drinking onset, and lifetime alcohol consumption in the post-mortem human orbitofrontal cortex (OFC) of age-matched moderate drinking control (CON) and alcohol use disorder (AUD) individuals. | | | |
| --- | --- | --- | --- |
|  | Fluoro-Jade B+IR | Age of Drinking Onset | Lifetime Alcohol Consumption (kg) |
| Fluoro-Jade B+IR |  | -0.88 ** | 0.74 ** |
| TLR9+IR | **0.84 **** | -0.88 ** | 0.63 ** |
| CXCL8+IR | **0.80 **** | -0.83 ** | 0.67 ** |
| CCL2+IR | **0.76 **** | -0.79 ** | 0.71 ** |
| MyD88+IR | **0.76 **** | -0.82 ** | 0.49 * |
| pRELA+IR | **0.75 **** | -0.66 ** | 0.41 |
| *TLR4* | **0.72 **** | -0.49 * | 0.86 ** |
| *CCL7* | **0.69 **** | -0.60 ** | 0.31 |
| *NFKB1* | **0.66 *** | -0.63 ** | 0.50 * |
| *TLR7* | **0.66 *** | -0.65 ** | 0.45 |
| Cleaved IL-1β+IR | **0.65 *** | -0.78 ** | 0.47 * |
| *TNFA* | 0.61 * | -0.68 ** | 0.12 |
| IKKβ+IR | 0.59 * | -0.80 ** | 0.30 |
| *CXCR4* | 0.59 * | -0.54 * | 0.22 |
| *CCL8* | 0.58 * | -0.56 * | 0.13 |
| *HMGB1* | 0.57 * | -0.57 ** | 0.29 |
| Pearson's r correlations assessed the association of induced neuroimmune genes with Fluoro-Jade B+IR, age of drinking onset, and lifetime alcohol consumption in post-mortem human OFC tissue samples from CON and AUD subjects. The Fluoro-Jade B+IR was reported previously (Qin and Crews, 2012b)The Benjamini-Hochberg procedure (B-H Critical) for controlling false positives was calculated (Thissen et al., 2002) for neuroimmune correlations with Fluoro-Jade B+IR. An individual Pearson’s r correlation was considered statistically significant if the *p* value was less than the B-H Critical value (false discovery rate threshold = 0.1) as denoted in **bold**. Pearson's r correlation coefficients were used with two-tailed significance. * *p* < 0.05, ** *p* < 0.01. | | | |
